# Temperature‐Tuned Electrocatalytic Valorization of Levulinic Acid to γ‐Valerolactone or 4‐Hydroxyvaleric Acid over CuNi(Ru)/Graphene Nanowalls

**DOI:** 10.1002/cssc.202502403

**Published:** 2025-11-25

**Authors:** Pol Vilariño, Jordi Rigual‐Miret, Ghulam Farid, Stefanos Chaitoglou, Roger Amade, Elvira Gómez, Albert Serrà

**Affiliations:** ^1^ Grup d’Electrodeposició de Capes Primes i Nanoestructures (GE‐CPN) Departament de Ciència de Materials i Química Física Universitat de Barcelona Barcelona Spain; ^2^ Institute of Nanoscience and Nanotechnology (IN^2^UB) Universitat de Barcelona Barcelona Spain; ^3^ Department of Applied Physics Universitat de Barcelona Barcelona Spain

**Keywords:** 4‐hydroxyvaleric acid, electrocatalytic biomass valorization, gamma‐valerolactone, graphene nanowalls, levulinic acid hydrogenation

## Abstract

Temperature‐modulated electrocatalytic hydrogenation of levulinic acid (LA) to γ‐valerolactone (GVL) or 4‐hydroxyvaleric acid (HVA) was investigated over CuNi and CuNiRu catalysts electrodeposited onto vertically aligned graphene nanowalls. Systematic potential (–1.6 to –2.0 V vs. Ag|AgCl) and temperature (5°C–50°C) studies revealed a clear product switch: at 5°C all catalysts showed > 95% selectivity to HVA, whereas at 50°C GVL dominated. Among the compositional configurations, trimetallic CuNiRu (50 mC cm^−2^) achieved the highest performance, affording 96.6% LA conversion, 92.4% GVL yield, and 98.5% selectivity at 50°C with minimal Ru loading. The synergy between Ru sites (promoting hydrogen activation and lactonization) and the high‐roughness nanowall scaffold suppressed H_2_ evolution, minimized metal leaching (<1%), and delivered stable operation under ambient pressure. The system maintained performance over multiple cycles and preserved selectivity even under concentrated LA solutions, confirming architectural robustness. Faradaic efficiencies up to 89%, low energy consumption (~0.12  kWh  mol^−1^), and energy storage efficiencies > 70% underscore the viability of this system for direct electricity‐to‐fuel conversion. These temperature–potential insights establish a tuneable platform where low‐temperature operation yields HVA, whereas moderate temperatures (50°C) enable near‐quantitative GVL production.

## Graphical abstract

Temperature‐modulated electrocatalysis over CuNi(Ru)/graphene nanowalls (GNWs) enables tuneable biomass valorization of levulinic acid. Synergistic alloy–support interactions direct selective hydrogenation to γ‐valerolactone or 4‐hydroxyvaleric acid with high efficiency, stability, and energy performance.

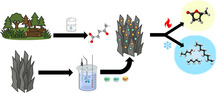

## 1 Introduction

The escalating climate crisis and the depletion of fossil fuel reserves demand a profound shift toward sustainable chemical manufacturing. Decarbonization and circular economy strategies are paramount to mitigate environmental impact and ensure long‐term resource resilience [1, 2, 3]. In this context, biomass, a renewable and abundant feedstock, emerges as a transformative solution, offering a pathway to substitute petroleum‐derived chemicals and foster a sustainable bioeconomy [4, 5]. This transition aligns seamlessly with the principles of green chemistry and contributes significantly to achieving crucial sustainable development goals by promoting environmentally friendly strategies for renewable chemical production [6].

Among biomass‐derived platform molecules, levulinic acid (LA)—readily obtained via lignocellulosic biomass depolymerization—has attracted considerable attention as a precursor to a wide array of value‐added chemicals. One of the most prominent derivate is gamma‐valerolactone (GVL), a compound with exceptional properties (i.e., nontoxicity, biodegradability, and high boiling point), making it an attractive sustainable solvent, fuel additive, and precursor for polymers and fine chemicals [5, 7, 8]. The catalytic conversion of LA to GVL holds significant market relevance due to the increasing demand for sustainable alternatives across various industrial sectors [9, 10].

In the electrocatalytic hydrogenation of LA, a key intermediate is 4‐hydroxyvaleric acid (HVA), which can also be obtained as a stable final product under specific conditions. HVA is a multifunctional molecule that retains a hydroxyl and a carboxyl group, rendering it useful for polymer synthesis and fine chemical production. Its high oxygen content and chemical reactivity also make it a potential platform compound in its own right. Importantly, HVA and GVL are closely connected through a thermally driven lactonization step, whereby HVA is converted to GVL via intramolecular esterification. This equilibrium is strongly influenced by reaction temperature, pH, and catalyst composition. Thus, understanding and controlling the HVA/GVL product distribution is crucial to optimizing selectivity for target applications—be it for direct use of HVA or for efficient production of GVL [11, 12, 13, 14].

Despite its promise, conventional LA‐to‐GVL hydrogenation often faces considerable drawbacks [15]. These methods typically demand harsh reaction conditions, including elevated temperatures and high pressures, necessitating external hydrogen sources derived from fossil fuels. Furthermore, the reliance on expensive and scarce noble metal catalysts, such as ruthenium or palladium, significantly increases process costs and environmental footprint. Consequently, there is a compelling need for a greener, inherently safer, and more tuneable alternative that can overcome these limitations, paving the way for more sustainable and economically viable GVL production [5, 16, 17].

Electrocatalysis emerges as a highly promising, low‐impact route for LA hydrogenation, offering a compelling alternative to traditional thermocatalytic processes [12, 18]. This innovative approach operates under significantly milder conditions, typically at ambient pressure and temperature, drastically reducing energy consumption and operational hazards [11]. Moreover, electrocatalysis can efficiently utilize aqueous solutions as a sustainable and abundant hydrogen source, eliminating the need for pressurized hydrogen gas. Crucially, the electrical control over the reaction allows for precise modulation of reaction rate and selectivity [19]. Despite these advantages, the exploration of aqueous electrocatalytic LA hydrogenation remains remarkably limited in the existing scientific literature [13, 14, 20].

While often considered a “room temperature” technique, temperature plays a critical yet largely unexplored role as a powerful control parameter in electrocatalysis [21, 22]. Temperature profoundly modulates reaction kinetics, influencing electron transfer rates, adsorption/desorption phenomena, and overall reaction pathways. It also impacts mass transport of reactants to the electrode surface, selectivity toward desired products, and the fundamental behavior of the electrode/electrolyte interface. In the context of LA reduction, temperature can shift the selectivity from HVA—favored at low temperatures due to its stability and the lower activation energy barrier for hydrogenation—to GVL, which requires additional activation energy for the lactonization step. Despite its multifaceted influence, few studies systematically explore temperature dependence in aqueous electrocatalytic biomass upgrading, especially concerning LA to GVL conversion. Therefore, this study undertakes a systematic investigation of thermal effects in electrocatalysis, focusing on GVL yield and efficiency [23].

This work strategically employs vertically aligned graphene nanowalls (GNWs) as a novel and efficient catalytic support. GNWs offer distinct advantages over conventional carbon‐based materials like carbon nanotubes (CNTs) and graphene [24, 25, 26, 27, 28, 29, 30]. Their unique three‐dimensional architecture provides a high density of exposed active sites and excellent electron conductivity, facilitating efficient charge transfer during electrocatalytic reactions [31, 32]. Furthermore, the open framework of GNWs promotes enhanced mass transport of reactants and products, minimizing diffusion limitations [27, 28, 33, 34]. Unlike the often entangled or stacked nature of CNTs and graphene, the vertically aligned structure of GNWs ensures superior accessibility and stability for the deposited catalysts [34, 35]. In contrast to entangled CNT networks and restacked rGO films, which often require additional functionalization to achieve sufficient metal anchoring and accessible porosity, vertically aligned GNWs intrinsically provide (i) a high density of exposed edge sites for homogeneous nucleation, (ii) an open three‐dimensional architecture that mitigates diffusion limitations, and (iii) intimate electronic coupling with the current collector. Previous studies have quantitatively demonstrated that GNW‐based electrodes exhibit higher electrochemically active surface area, improved mass transport, and stronger metal–support interactions than conventional carbon supports under comparable conditions [36, 37, 38, 39].

Our choice of Cu–Ni–(Ru) catalyst systems is grounded in a robust rationale. Copper (Cu) and nickel (Ni) are earth‐abundant and low‐cost metals, both recognized for their inherent hydrogenation activity, making them economically viable and sustainable catalytic components [29, 40, 41]. The strategic incorporation of ruthenium (Ru), even at low loadings, is designed to significantly enhance the overall catalytic activity, improve selectivity toward GVL, and bolster the long‐term stability of the catalyst [42, 43, 44]. Prior work has demonstrated the synergistic effects of combining these metals, where Ru can act as a promoter, facilitating hydrogen activation and enhancing the performance of the more abundant Cu and Ni [19].

Despite the clear potential of electrocatalytic LA hydrogenation, a significant gap exists in the literature regarding a systematic understanding of the interplay between catalyst composition, support morphology, and reaction temperature [45, 46]. Specifically, the influence of thermal effects on the efficiency and selectivity of this process, particularly when employing novel nanomaterials and earth‐abundant catalysts, remains largely unexplored. Therefore, the objective of this work is to evaluate the electrocatalytic conversion of LA to GVL using Cu–Ni–(Ru) catalysts supported on GNWs, with a focused investigation on the influence of reaction temperature on HVA and GVL selectivity, overall LA conversion, and catalyst performance.

## 2 Experimental Section

### 2.1 Synthesis and Characterization of Electrocatalysts

GNWs were grown on a Papyex flexible paper substrate in an inductively coupled plasma chemical vapor deposition (ICP‐CVD) system. A detailed description of the synthesis process can be found in the literature [26, 39, 47]. Papyex sheets (approximate dimensions 35 × 50 mm) were vigorously washed in acetone and deionized water to remove surface impurities and then dried under nitrogen atmosphere to ensure complete removal of residual moisture. The substrate samples thus purified were clamped on a substrate holder consisting of a piece of graphite and loaded in the ICP‐CVD reactor chamber.

The ICP‐CVD system consists of a long quartz tube, a radio‐frequency resonator (13.56 MHz, 440 W) for the formation of remote plasma and an external tubular furnace for temperature control. The chamber was evacuated to a base pressure in the order of 10^−4^ mTorr using a turbomolecular pump for a contaminant‐free area. The substrate was placed 30 cm away from the plasma production zone to exclude direct ion bombardment and was heated to 750°C.

The growth process was triggered by an initial surface activation step: a hydrogen plasma (H_2_ flow at 15 sccm) was applied at 400 W in a pressure of 400 mTorr for 5 min. This step allows cleaning of residual contaminants as well as substrate surface activation for proper carbon nucleation. The flow of H_2_ was then closed and methane (CH_4_) gas was introduced at a flow of 10 sccm. Under identical plasma settings (400 W, 400 mTorr), the CH_4_ plasma was driven for 30 min, causing GNWs growth on the substrate surface. Afterward, the substrate was let to cool down to ambient temperature in a vacuum atmosphere to suppress unwanted oxidation. Surface hydrophilicity and wettability of the GNWs were enhanced by a final oxygen plasma treatment. The sample was treated for 30 s in O_2_ plasma (13 sccm) at an RF power value of 40 W and 400 mTorr pressure.

Two freshly prepared aqueous solutions were formulated for the electrodeposition of CuNi and CuNiRu electrocatalysts, as detailed in Table S1. The precursors salts used were NiCl_2_·6H_2_O (Merck, 99.9%, CAS: 7791‐20‐0), CuCl_2_·2H_2_O (Sigma–Aldrich, >99.0%, CAS: 10125‐13‐0), RuCl_3_·3H_2_O (Sigma–Aldrich, >99.0%, CAS: 13815‐94‐6), and HOC(COONa)(CH_2_COONa)_2_·2H_2_O (Sigma–Aldrich, >99.0%, CAS: 6132‐04‐3). NaCl (Sigma–Aldrich, >99.0%, CAS: 7647‐14‐5) was added to regulate the ionic strength, which was adjusted to 2.30 M for both solutions to ensure uniform conditions. Prior to each experiment, the pH was adjusted to 3.0 using hydrochloric acid, and all solutions were prepared using deionized Milli‐Q water. Electrodeposition experiments were conducted at a constant temperature of 25°C.

The metal ion concentrations were tailored to obtain Ni‐rich electrocatalysts. Nickel (II) concentration was present at 15‐fold the molar concentration of ruthenium (III) and 6‐fold that of copper (II). This high Ni content was selected despite the overlap of its reduction with hydrogen evolution, due to its favorable cost and known electrochemical stability. The Ru(III) concentration was minimized to reduce cost while still enabling its function as a catalytic promoter.

Electrochemical measurements were carried out using a Metrohm Autolab PGSTAT204 potentiostat/galvanostat, controlled via NOVA 2.1 software. A conventional three‐electrode configuration setup was utilized, consisting of a platinum wire as the counter electrode, an Ag|AgCl|Cl^−^ (3.0 M) electrode as a reference, and either a glassy carbon electrode (GC) or vertically aligned GNWs as the working electrode. Prior to each experiment, the GC electrode was polished to a mirror finish using alumina powders of decreasing particle size (3.75 and 1.87 µm) on polishing pads. After the polishing step, the electrode was rinsed with deionized water to remove any residual alumina particles. It was then sonicated in Milli‐Q water for 2 min to dislodge any remaining impurities. All electrochemical experiments were performed under an argon atmosphere. Cyclic voltammetry (CV) was used to analyze the electrochemical behavior in each bath, while potentiostatic chronoamperometry (CA) was employed for electrodeposit preparation.

The morphology and elemental composition of the electrodeposited films were examined by field‐emission scanning electron microscopy (FE‐SEM; JEOL‐7100) equipped with energy‐dispersive X‐ray spectroscopy (EDS). Quantification of the elemental content and the total metal loading was performed using inductively coupled plasma optical emission spectrometry (ICP‐OES; Optima 8300, PerkinElmer) for ppm‐level analysis, and inductively coupled plasma mass spectrometry (ICP‐MS; NexIon 2000, PerkinElmer) for ppb‐level detection, enabling evaluation of Faradaic efficiency. Crystalline phase identification was carried out by X‐ray diffraction (XRD) using a PANalytical X’Pert PRO MPD Alpha1 diffractometer with monochromatic Cu Kα radiation (*λ *= 1.5418 Å). Data were collected in *θ*–2*θ* geometry over a 2*θ* range of 4.5°–100°, with a step size of 0.026° and a dwell time of 200 s per step; three scans were recorded for each sample. Surface chemical composition was analyzed by X‐ray photoelectron spectroscopy (XPS; PHI Quantera SXM) under ultra‐high vacuum using a monochromatic Al Kα source.

### 2.2 Electrocatalytic Reduction of LA

The electrochemical reduction of LA was performed using the same potentiostat–galvanostat system (Metrohm Autolab PGSTAT204) and three‐electrode configuration described in Section 2.1. Vertically aligned GNWs or GNWs modified by electrodeposited metals were used as the working electrode, with an Ag|AgCl|Cl^−^ (3.0 M) electrode as reference and a platinum spiral as counter electrode. The electrochemical medium consisted of an acidic aqueous solution containing 0.5 M LA and 0.5 M KCl as supporting electrolyte, adjusted to pH ≈ 0 with H_2_SO_4_, as detailed in Table S2. All experiments were conducted using 15 mL of electrolyte solution.

Temperature‐dependent studies were conducted at 5°C, 15°C, 25°C, 35°C, and 50°C to evaluate the thermal effects on catalytic performance. Linear sweep voltammetry (LSV) was carried out in the absence and presence of LA at 50 mV s^−1^ to assess the electrochemical response and determine suitable potentials for the electrocatalytic hydrogenation. Potentiostatic CA was performed at fixed potentials until a total charge of 1500 C was passed. The choice of applied potentials was guided by LSV results. After electrolysis, the electrolyte solutions were filtered and analyzed to quantify LA conversion and identify products.

The total organic carbon (TOC) content in solution was quantified by high‐temperature combustion using a TOC‐V_CSH_ analyzer (Shimadzu) to estimate the total amount of nonvolatile organic products remaining in the electrolyte after electrolysis. As volatile organic compounds (VOCs) are not retained in solution and thus not detected by TOC analysis, a reduction in TOC may indicate the formation of volatile byproducts. Product distribution was further analyzed by high‐performance liquid chromatography (HPLC) with UV–vis detection at 210 nm. GVL was quantified using a reverse‐phase XBridge C18 column (3.5 μm, 4.5 × 50 mm) with isocratic elution of water: acetonitrile (9:1, v/v) and a 10 μL injection volume. LA, VA, and 4‐HVA were analyzed using an Aminex HPX‐87H column (300 × 7.8 mm) at 60°C, with 10 mM H_2_SO_4_ in water: acetonitrile (9:1, v/v) as the mobile phase and a 50 μL injection volume.

^1^H nuclear magnetic resonance (NMR) spectra were acquired using Bruker 400 MHz and Bruker 500 MHz. Chemical shifts (δ) are reported in parts per million (ppm), referencing either tetramethylsilane (TMS) or the residual solvent signal (chloroform, 1H at 7.26 ppm) as internal standards. Coupling constants (*J*) are expressed in hertz, and spectrum processing was conducted using MestreNovaR. Signal multiplicities are denoted by standard abbreviations: s (singlet), d (doublet), t (triplet), q (quartet), and m (multiplet). The reaction mixture was extracted with ethyl acetate (1:1, v/v), and the organic phase was subsequently washed 3 times with 10 mL portions of 10% aqueous sodium bicarbonate (NaHCO_3_) to neutralize residual acidity and remove water‐soluble impurities. This procedure was employed to purify the final product, GVL, prior to further analysis.

Catalyst stability and scalability were further evaluated using the best‐performing configuration. Five consecutive potentiostatic electrolysis cycles were performed at 50°C in an aqueous solution containing 0.5 M LA and 0.5 M KCl, adjusted to pH ≈ 0 with H_2_SO_4_. For each cycle, 15 mL of fresh electrolyte was used and the functionalized electrode was polarized at the selected potential until a total charge of 1500 C was passed. After each run, the electrolyte was collected for HPLC analysis and TOC determination, and the electrode was gently rinsed with deionized water and immediately reused without any mechanical or electrochemical regeneration. The liquid phase after each cycle was also analyzed by ICP‐OES following the same procedure described previously to quantify possible Cu, Ni, and Ru leaching.

In addition, to evaluate catalyst performance under more demanding conditions, a concentrated solution experiment was carried out at 50°C in 15 mL of 4.0 M LA + 0.5 M KCl (pH ≈ 0, H_2_SO_4_). Potentiostatic electrolysis was performed at the same potential, passing a total charge of 12 000 C to keep a comparable charge‐to‐substrate ratio. Postelectrolysis solutions were analzsed by HPLC, TOC, and ICP‐OES as above. No cleaning or reactivation of the electrode was applied prior to this test beyond gentle rinsing with deionized water. For the most effective material and reaction conditions, postelectrolysis characterization was performed. In selected cases, FE‐SEM analysis was carried out to examine morphological changes in the electrocatalyst surface after electrolysis.

## 3 Results and Discussion

### 3.1 Synthesis and Characterization of Electrocatalysts

GNWs exhibit an electrochemically active surface area that is one to two orders of magnitude greater than that of planar GC [48]. When the previously reported bath composition optimized for flat GC—0.30 M Ni(II), 0.05 M Cu(II), and 0.02 M Ru(III)—was applied to GNWs, CV revealed a proportional increase in geometric current density consistent with the enlarged surface area. This enhancement led rapidly to mass‐transport‐limited conditions and uncontrolled metal deposition, even under stirring (Figure S1), complicating the control of film composition and morphology [19]. To adapt the deposition process to the high surface area of GNWs, the concentrations of Ni(II), Cu(II), and Ru(III) were reduced by one order of magnitude. This adjustment decreased the flux of metal ions to the electrode, lowering the overall deposition rate and minimizing the impact of differences in metal ion reduction kinetics and diffusion coefficients. As a result, the relative deposition rates of the individual metals became more balanced, favoring the formation of compositionally uniform, Ni‐rich coatings. In addition, the lower current densities helped suppress the competitive hydrogen evolution reaction (HER), improving the quality and adherence of the films. The resulting CuNi and CuNiRu coatings displayed Faradaic efficiencies and elemental compositions comparable to those obtained on planar GC under optimized conditions.

**FIGURE 1:**
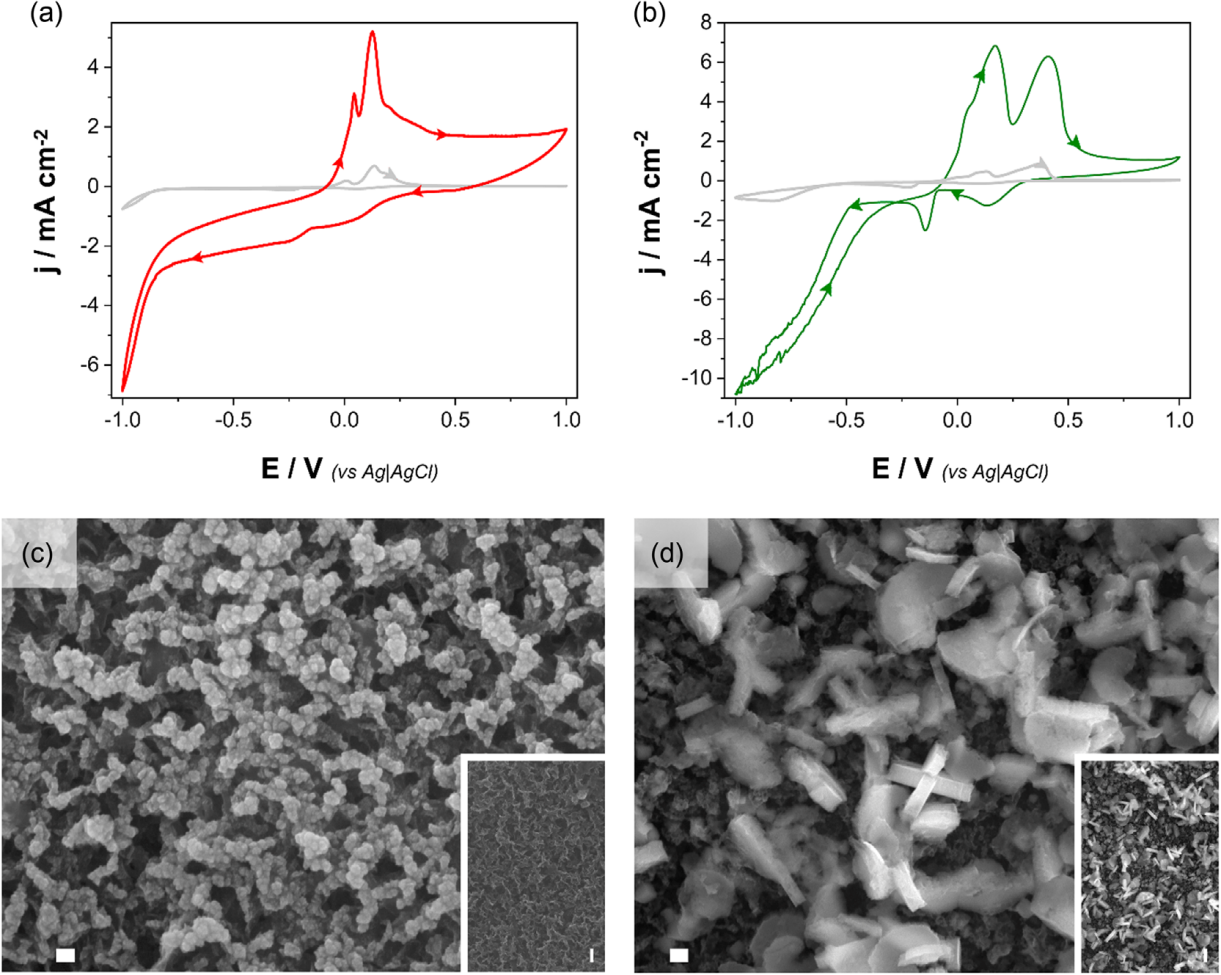
CV profiles of the (a) CuNi electrolyte and (b) CuNiRu electrolyte recorded on a glassy carbon electrode (gray curve) and a GNW‐modified electrode, with a scan rate of 50 mV s^−1^ at 25°C. The voltammograms illustrate the influence of the electrode substrate on metal deposition behavior and the onset of the HER. FE‐SEM image of the (c) CuNi deposit and (d) CuNiRu deposit on GNWs. Both deposits were prepared potentiostatically at –1.0 V versus Ag|AgCl (deposition charge density: 50 mC cm^−2^). Scale bar: 200 nm.

As shown in Figure 1, the cyclic voltammograms of CuNi and CuNiRu baths recorded on GC and GNW electrodes reveal a substantial enhancement in current density for the GNWs, consistent with their significantly larger electrochemically active surface area. In the CuNi system (Figure 1a), several reduction features are shown. The first cathodic signal, appearing as a broad shoulder centered around + 0.25 V, corresponds to the reduction of Cu(II) to Cu(I). A second, more intense cathodic peak, located at approximately −0.15 V, is attributed to the subsequent reduction of Cu(I) to metallic Cu(0). At more negative potentials, below approximately −0.80 V, the onset of Ni(II) reduction is observed. Beyond −0.90 V, a sharp increase in cathodic current occurs, reflecting the concurrent reduction of Ni(II), Cu(I/II), and protons, characteristic of the codeposition regime and HER [49]. The anodic scan exhibits two distinct oxidation features. The first peak, centered at lower potentials, is primarily attributed to the oxidation of Cu(0) to Cu(I), with possible contribution from the partial oxidation of Ni(0) to α‐Ni. The second, broader oxidation peak at higher potentials is associated with the Cu(I) → Cu(II) transformation and partial stripping of β‐Ni or surface‐passivated nickel species [50, 51, 52]. Notably, when stirring is applied during the anodic scan, the intensity of this second peak is significantly diminished, suggesting that a non‐negligible portion of the current arises from the homogeneous oxidation of dissolved Cu(I) to Cu(II).

In the CuNiRu bath (Figure 1b), the CV in the cathodic scanning, the reduction of Cu(II) to Cu(I) is first observed, manifested as a moderate cathodic peak in the positive potential region, at +0.10 V. As the potential continues to decrease, the reduction of Cu(I) to Cu(0) occurs, generating a second, more negative peak at, approximately −0.25 V, corresponding to the deposition of metallic copper on the electrode. Subsequently, another reduction signal is observed at −0.40 V, corresponding to the process that involves the reduction of Ru(III) to Ru(0) [53, 54]. Finally, at more negative potentials, close to −1.00 V, the reduction of nickel (Ni(II) to Ni(0) is identified, followed/overlapped by the beginning of the HER, evidenced by an abrupt increase in the cathodic current, indicative of the generation of H_2_ in the electrode. During the subsequent anodic scan, multiple overlapping oxidation peaks are observed, reflecting the complex reoxidation behavior of the deposited metals.

The surface morphologies of pristine and metal‐modified GNWs were investigated by FE‐SEM. The electrodeposition of all metallic deposits was carried out at a potential of –1.0 V, as determined through a CV study. As illustrated in Figure S2, pristine GNWs exhibit a morphology characterized by a dense forest of vertically aligned, sheet‐like graphene structures. These nanowalls manifest sharp edges, elevated aspect ratios, and considerable interwall spacing, thereby establishing an open, three‐dimensional, and interconnected architecture. This configuration provides a substantial electrochemically active surface area, thereby rendering GNWs a highly suitable template for metal electrodeposition.

When a low deposition charge of 25 mC cm^−2^ was employed, CuNi deposition on GNWs (Figure S3a) resulted in the formation of nanoscale metallic clusters. These clusters were preferentially located along the edges and exposed facets of the nanowalls. This observation indicated the occurrence of heterogeneous nucleation at high‐energy surface sites. At this stage, the deposition remained discontinuous, thereby allowing the underlying GNW texture to remain clearly visible. The incorporation of ruthenium in the ternary CuNiRu system led to notable changes in morphology, as seen in Figure S3b. At the same deposition charge (25 mC cm^−2^), a higher density of finer nanoparticles was observed, with a more uniform distribution across the GNW surface. The presence of Ru was observed to have a twofold effect: it enhanced nucleation density and promoted a more homogeneous dispersion of the metallic phase.

A higher deposition charge of 50 mC cm^−2^ resulted in the observation of a greater number of continuous and granular coatings. In the CuNi system (Figure 1c), the metal layer demonstrates substantial growth, with agglomerated particles coalescing and enveloping a significant portion of the GNW surface. While the vertical alignment of the nanowalls remains partially discernible, the overall texture becomes increasingly obscured due to the thickening of the deposit. Conversely, the CuNiRu deposit at 50 mC cm^−2^ (Figure 1d) formed a denser and more conforming coating. The GNWs were found to be predominantly encapsulated within a compact network of uniformly distributed nanoparticles interspersed with nanowire‐ or nanocolumn‐like features, resulting in a smoother and more homogeneous morphology. This finding indicates that Ru plays a pivotal role in regulating the microstructure of the resulting trimetallic coatings, facilitating not only enhanced nucleation but also vertical growth and improved film densification.

EDS analysis further corroborated the morphological observations from FE‐SEM, confirming the successful co‐deposition of Cu, Ni, and Ru with uniform elemental distribution. In the bimetallic CuNi system, the coatings displayed a Ni‐rich composition of approximately 14 at.% Cu and 86 at.% Ni, reflecting the preferential reduction of Ni under the selected conditions. In the trimetallic CuNiRu system, the incorporation of Ru was clearly evidenced, with a homogeneous composition of approximately 20 at.% Cu, 13 at.% Ru, and 67 at.% Ni. These results indicate that the deposition protocol yields compositionally consistent and well‐integrated bimetallic and trimetallic films.

The XRD patterns of CuNi‐ and CuNiRu‐decorated GNWs (Figure S4) reveal multiple diffraction peaks that offer insight into the structural characteristics of the hybrid materials. Based on the literature, the diffractogram of the Papyex paper exhibits various peaks indicative of its polycrystalline nature. Several reflections are also associated with the carbonaceous scaffold [47, 55]. In particular, the peak at 26.58° (2*θ*) corresponds to the (002) plane of graphitic carbon (JCPDS 00‐001‐0640), characteristic of graphene‐based materials. Additional peaks at 54.66°, 78.06°, and 86.93° can be assigned to higher order graphitic reflections, such as the (004), (110), and (006) planes, respectively, commonly observed in well‐ordered or stacked graphene structures. The presence of these signals confirms the structural integrity of the GNWs following metal deposition [26, 47, 56].

For the CuNi sample, a prominent diffraction peak at 44.49° (2*θ*) can be indexed to the (111) plane of a face‐centered‐cubic (FCC) CuNi system. Its proximity to the (111) reflection of pure Ni (44.5°) and its slight shift from that of pure Cu (43.3°) indicate a Ni‐rich alloy composition, consistent with the electrolyte formulation and deposition conditions. Additional reflections at 78.06°–78.29° and 98.94°–99.27° are assigned to the (311) and (400) planes of the FCC structure, further supporting the formation of a well‐crystallized bimetallic phase. Minor peaks at 38.30° and 65.11° may be attributed to residual or surface‐formed oxide species, such as NiO or Cu_2_O, possibly due to postdeposition air exposure.

The CuNiRu sample exhibited a similar pattern, with several additional reflections. A notable diffraction peak at 44.37° (2*θ*) corresponds to the (111) plane of a CuNiRu‐based FCC solid solution. Compared to the CuNi sample, the slight shift to lower angles suggests incorporation of Ru into the CuNi lattice, leading to a small expansion of the unit cell and indicative of lattice distortion caused by Ru atoms. Additional peaks at 54.62°, 64.77°, 77.54°, and 86.99° correspond to the (200), (220), (311), and (222) reflections of the FCC structure, confirming the retention of crystallinity upon Ru incorporation. Weaker reflections at 23.89°, 29.54°, and 36.31° may originate from turbostratic or disordered carbon, or trace Ru‐based secondary phases, such as RuO_2_ or segregated Ru clusters. Peaks at 42.26° and 61.28°, which do not correspond to typical FCC reflections, could be attributed to intermetallic phases or crystalline Ru‐containing species that are not fully incorporated into the alloy matrix.

Overall, the XRD analysis confirms the successful electrodeposition of crystalline CuNi and CuNiRu alloys on GNWs. The preservation of the graphitic features and the formation of well‐defined alloy phases—along with partial Ru incorporation and the potential presence of minor secondary phases—highlight the structural complexity and robustness of the synthesized hybrid catalysts.

XPS analysis was employed to investigate the chemical composition and oxidation states of the deposited materials. As shown in Figure S5a**,** the Cu 2*p* XPS spectrum of the CuNi system displayed characteristic peaks at 933.2 and 952.9 eV, corresponding to the Cu 2*p*_3/2_ and Cu 2*p*_1/2_ core levels of metallic copper (Cu), respectively. Additionally, weaker peaks at 935.2 and 954.4 eV were observed, along with satellite features around 943, 945, and 964 eV, which are indicative of Cu(II) species. These Cu(II) signals likely arise from partial surface oxidation upon exposure to air. A similar Cu 2*p* spectral profile was observed for the CuNiRu system (Figure S5a). For both CuNi and CuNiRu samples (Figure S5b), the Ni 2*p*_3/2_ XPS spectra featured a main peak at 852.5 eV, characteristic of metallic nickel (Ni^0^), along with two prominent satellite peaks at 856.3 and 858.6 eV. Minor contributions from nickel oxides and hydroxides were also detected, as evidenced by small additional features in the spectra. The O 1*s* spectra of both deposits showed similar profiles, with a peak at ~531.5 eV attributed to metal–oxygen bonds and a second peak at ~532.8 eV corresponding to surface hydroxyl (—OH) groups. Notably, the hydroxyl contribution was more pronounced in the CuNiRu sample. Due to overlap with the intense C 1*s* signal, the Ru 3*d* region was not clearly resolved. Therefore, the Ru 3*p* region was analyzed instead (Figure S5d). The Ru 3*p* spectrum exhibited spin–orbit splitting into Ru 3*p*_3/2_ and Ru 3*p*_1/2_ components. Peaks at 462.1 and 465.7 eV in the Ru 3*p*_3/2_ region were assigned to Ru(0) and RuO_2_, respectively. Similarly, peaks at 484.3 and 486.5 eV in the Ru 3*p*_1/2_ region were attributed to the same species. The dominant Ru species was metallic Ru(0), confirming the successful incorporation of Ru in its reduced form [57].

### 3.2 Electrocatalytic Conversion of LA to High Value Chemicals

**FIGURE 2:**
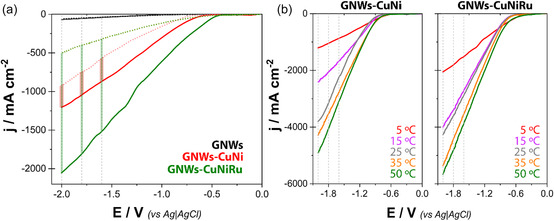
(a) LSVs recorded from 0.0 to –2.0 V versus Ag|AgCl|Cl^−^ at a scan rate of 50 mV s^−1^ in the absence (dashed lines) and presence (solid lines) of 0.5 M LA, using GNWs, CuNi (25 mC cm^−2^), and CuNiRu (25 mC cm^−2^) electrodes at 5°C. (b) LSVs recorded under identical conditions (0.5 M LA, 50 mV s^−1^ scan rate, 0.0 to –2.0 V vs Ag|AgCl|Cl^−^) for CuNi and CuNiRu electrodes (25 mC cm^−2^) at different temperatures: 5°C, 15°C, 25°C, 35°C, and 50°C.

The electrochemical reduction of LA was investigated by LSV using GNWs, CuNi, and CuNiRu electrodes under varying conditions of potential and temperature. For clarity, the catalyst labels used throughout this work follow the notation CuNi–X and CuNiRu–X, where X represents the total deposition charge in mC cm^−2^. Specifically, CuNi–25 and CuNi–50 correspond to bimetallic CuNi films deposited at 25 and 50 mC cm^−2^, respectively, while CuNiRu–25 and CuNiRu–50 denote the analogous trimetallic CuNiRu coatings. In the absence of LA, the cathodic current was primarily attributed to the HER, and the response was largely governed by the HER activity on the respective materials. Upon the incorporation of 0.5 M LA (Figure 2a), each system displayed different degrees of current enhancement, reflecting their catalytic ability to engage in LA reduction.

GNWs exhibited marginal changes in current density with or without LA (–37.3 vs. –40.1 mA cm^−2^ at –1.6 V), confirming that the carbon support alone is catalytically inert toward LA reduction. However, the GNW scaffold plays a pivotal structural role in determining catalyst performance. Its vertical, open architecture enables high metal dispersion and short electron pathways while maintaining unobstructed channels for electrolyte penetration and product removal. In contrast, the CuNi system displayed a more substantial increase, from –573.2 to –872.7 mA cm^−2^ under the same conditions—representing a 52% enhancement. This modest improvement suggests that the bimetallic CuNi interface provides active sites for LA adsorption and facilitates proton‐coupled electron transfer, though the overall selectivity remains partially limited by competing HER.

The most dramatic effect was observed for the CuNiRu electrode, where current densities increased from –323.7 to –1521.8 mA cm^−2^ at –1.6 V upon LA addition—a nearly fivefold increase. This substantial boost not only reflects enhanced intrinsic activity but also a significant shift in electron allocation from HER toward LA hydrogenation. The enhancement factor remained high across the potential range examined, although it declined slightly at more negative potentials (from 4.7 at –1.6 V to 4.1 at –2.0 V), indicating that while the catalytic benefit of Ru remains dominant, increasing overpotential promotes a rise in HER competition. These trends underscore the pivotal role of Ru as a cocatalyst. Ru is well known for its ability to dissociate protons and generate adsorbed hydrogen efficiently. In this context, its incorporation likely enhances hydrogen spillover onto the CuNi matrix, where LA molecules adsorbed on oxophilic Ni and Cu sites undergo rapid hydrogenation. The result is a synergistic mechanism in which Ru facilitates the initial hydrogen activation, while Cu and Ni modulate LA binding and reactivity. This cooperative effect is particularly evident at lower potentials, where reaction kinetics dominate over mass transport, and Ru effectively lowers the energy barrier for the hydrogenation of the carbonyl moiety in LA.

To further elucidate this behavior, temperature‐dependent LSV measurements were performed in the presence of 0.5 M LA (Figure 2b). For both CuNi and CuNiRu, current densities increased significantly with temperature at all potentials, consistent with enhanced reaction kinetics and mass transport. For instance, at –2.0 V, the current density on CuNi increased from –1203.6 mA cm^−2^ at 5°C to –4904.1 mA cm^−2^ at 50°C—a 4.1‐fold increase. CuNiRu displayed a comparable trend, rising from –2055.4 to –5658.7 mA cm^−2^ over the same temperature range (2.8‐fold increase).

Interestingly, the relative advantage of CuNiRu over CuNi decreased with temperature. At 5°C and –1.6 V, CuNiRu outperformed CuNi by a factor of 1.74, while at 50°C this ratio declined to 1.25. This suggests that the apparent activation energy for LA reduction is lower on CuNiRu, rendering it more effective at low temperatures. However, as temperature increases, thermal energy compensates for the higher activation barrier on CuNi, leading to performance convergence. This behavior highlights the role of Ru in thermally decoupling the electrocatalytic activity from temperature—an advantage in low‐energy or ambient‐temperature applications.

Moreover, the potential dependence of current enhancement provides further mechanistic insights. For both catalysts, the rise in current density with increasing cathodic potential reflects improved thermodynamic driving force. However, the diminishing enhancement factor with potential—particularly for CuNiRu—indicates that at high potentials (e.g., –2.0 V), the surface becomes increasingly saturated with adsorbed *H and HER prevails. In this regime, LA hydrogenation competes less favorably, resulting in a reduced benefit from Ru co‐catalysis. Therefore, CuNiRu could achieve its maximum selective advantage at intermediate potentials, such as –1.6 to –1.8 V, where HER is not yet dominant and kinetic effects control the current.

These findings have practical relevance for designing electrocatalysts for LA conversion to GVL, HVA, and other value‐added products. While both CuNi and CuNiRu exhibit high activity at elevated temperatures and potentials, the Ru‐doped system stands out under mild conditions, offering significant current enhancement (e.g., –1.5 A cm^−2^ at –1.6 V and 5°C) with relatively low catalyst loading. This is particularly attractive for decentralized biomass valorization processes operating at low energy input. In summary, CuNiRu electrodes exhibit superior catalytic activity for LA electroreduction due to the synergistic interplay between Ru‐mediated *H generation and Cu/Ni‐driven substrate activation. The enhancement is most pronounced under kinetic control (low T and mild overpotential), while diffusion limitations at higher driving forces level the performance gap.

**FIGURE 3:**
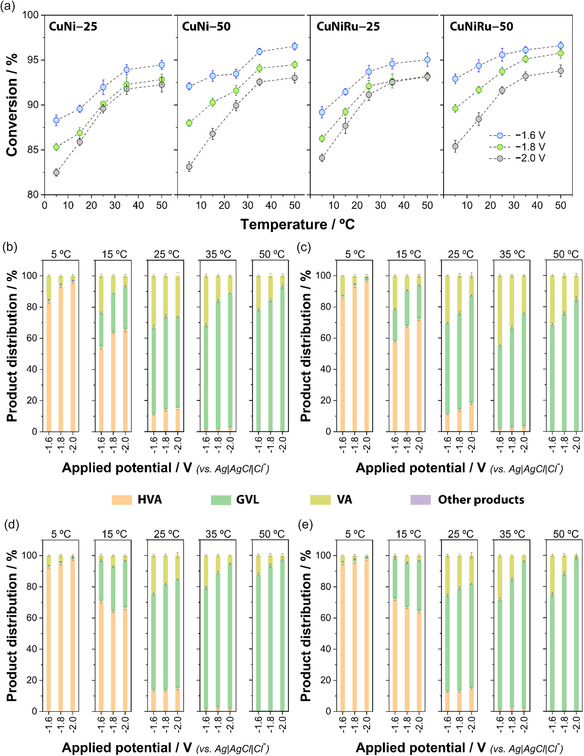
(a) LA conversion as a function of temperature for CuNi and CuNiRu catalysts electrodeposited on GNWs (25 and 50 mC cm^−2^), evaluated at three applied potentials (–1.6, –1.8, and –2.0 V vs. Ag|AgCl). (b–e) Molar product distribution as a function of applied potential for each catalyst at selected temperatures: (b) CuNi–25 mC cm^−2^, (c) CuNi–50 mC cm^−2^, (d) CuNiRu–25 mC cm^−2^, and (e) CuNiRu–50 mC cm^−2^. GVL, γ‐valerolactone; HVA, 4‐hydroxyvaleric acid; VA, valeric acid. Results are presented as mean ± standard deviation from triplicate experiments.

Figure 3a presents the temperature‐dependent conversion of LA for four GNW‐supported electrocatalysts—bimetallic CuNi and trimetallic CuNiRu—prepared at different metal loadings (25 and 50 mC cm^−2^) and evaluated at three applied potentials (–1.6, –1.8, and –2.0 V). For all catalyst–potential combination, the LA conversion increased monotonically from 5°C to 50°C, demonstrating a strong thermal enhancement of the electrocatalytic process. Even under the least active condition (CuNi 25 mC cm^−2^, −2.0 V) conversion rose from ∼82% at 5°C to ∼92% at 50°C, whereas the most active configuration (CuNiRu 50 mC cm^−2^, −1.6 V) achieved ∼93% conversion at 5°C and ∼97% at 50°C.

At all temperatures the hierarchy −1.6 V > −1.8 V > −2.0 V was preserved, indicating that a milder cathodic bias favors selective LA reduction over the competing HER. The penalty for operating at −2.0 V instead of −1.6 V was most severe at low temperature (∼6 percentage points at 5°C) and diminished to ≤ 2 points at 50°C, implying that HER becomes less competitive once diffusion and surface kinetics are thermally accelerated. Doubling the deposited charge from 25 to 50 mC cm^−2^ yielded a mean conversion increment of 2–3 percentage points at −1.6 and −1.8 V, but < 1 point at −2.0 V. The benefit of higher loading is therefore limited to regimes in which the productive hydrogenation pathway is not already starved of electrons by HER. Incorporation of Ru into the bimetallic CuNi matrix consistently enhanced performance by 1–2 percentage points under identical loading and bias, the effect being most pronounced at 15°C and −1.6 V. The improvement is attributed to the well‐documented ability of Ru to expedite hydrogen activation and to stabilize hydrogenated intermediates, thereby complementing the Cu‐ and Ni‐centered reduction sites.

Overall, the best‐performing system was CuNiRu–50 operated at –1.6 V and 50°C, achieving ∼97% LA conversion. Notably, this catalyst also exhibited high conversion at 5°C (∼93%), confirming its potential for efficient operation under mild thermal conditions. These results indicate that optimal performance arises from the synergistic combination of (i) moderate reductive bias to suppress HER, (ii) increased metal loading to enhance active surface area, and (iii) strategic Ru incorporation to promote catalytic activity [58, 59, 60].

Prior to determining the product distribution, TOC measurements were performed to assess the potential formation of VOCs during the electrochemical reduction of LA. The TOC values remained virtually unchanged compared to the initial concentration of the fresh LA solution, indicating that the overall carbon balance was preserved. At low temperatures (≤15°C), TOC decreased by less than 6%, while at higher temperatures (≥25°C), the reduction was negligible (<1%), confirming the absence of significant volatilization or degradation. These findings are consistent with the physicochemical properties of the expected products—GVL, HVA, and valeric acid (VA)—which exhibit high boiling points (>186°C) and extremely low vapor pressures and Henry constants (≤3 × 10^−6^ atm m^3^ mol^−1^). Such characteristics render them nonvolatile under the operating conditions applied (acidic aqueous electrolyte, 5°C–50°C, –1.6 to –2.0 V) [61]. Furthermore, literature reports confirm that reductive cleavage pathways leading to genuinely volatile products (e.g., C_3_–C_4_ ketones or aldehydes) are not favored in acidic, low‐temperature electrochemical systems and are typically only observed under high‐temperature oxidative conditions (≥180°C). Taken together, these results demonstrate that the electrocatalytic LA hydrogenation pathway employed here proceeds with negligible formation of VOCs, which is a notable environmental advantage over thermochemical or oxidative valorization strategies.

Figure 3b–e details the product distribution obtained during the electrochemical hydrogenation of LA at different potentials, temperatures, and catalyst configurations. The primary reaction products were GVL, HVA, and VA, while minor side products (<1%) were collectively grouped as “others.” These traces were below the detection limit of our NMR and HPLC methods and exhibited no identifiable peaks in GC analyses; therefore, no assignable byproducts were detected, and their negligible contribution does not influence the reaction pathway or safety considerations. A distinct temperature‐dependent shift in selectivity was observed across all systems, indicating that temperature plays a pivotal role in steering the overall reaction pathway.

**FIGURE 4:**
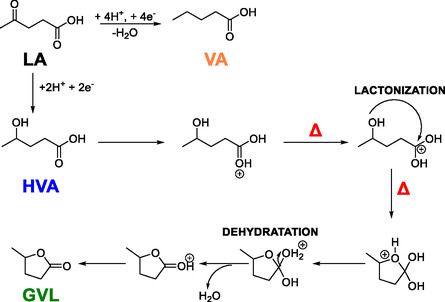
Proposed mechanism for the aqueous‐phase electrocatalytic conversion of LA over CuNi‐ and CuNiRu‐based cathodes. The reaction begins with a two‐electron, two‐proton electrochemical hydrogenation of the carbonyl group in LA, yielding 4‐hydroxypentanoic acid (HVA). From this intermediate, two competing pathways emerge: (i) a thermally activated intramolecular lactonization (denoted by Δ) in which the pendant hydroxyl group attacks the carboxylic acid moiety, forming a protonated lactone intermediate. This is followed by dehydration, also temperature‐dependent, to generate GVL as the target product; and (ii) under more cathodic conditions, a competing C—O bond hydrogenolysis—via an additional two‐electron, two‐proton transfer—leads to the formation of VA, representing an over‐hydrogenation pathway.Gray arrows indicate electrochemical (potential‐driven) steps, while red Δ symbols denote purely thermal, chemical transformations. This mechanistic scheme accounts for the observed temperature‐dependent product distribution: HVA dominates at low temperature due to sluggish lactonization, whereas GVL becomes the major product at ≥35°C. The transient formation of VA at intermediate temperatures is consistent with kinetic competition between ring closure and reductive hydrogenolysis.

At low temperatures (5°C–15°C), HVA was consistently the dominant product, regardless of catalyst composition or applied potential. This trend suggests that the initial hydrogenation of LA to HVA occurs readily under all tested conditions, while subsequent transformation to GVL is kinetically hindered. As the temperature increased to 35°C and above, a significant shift toward GVL selectivity emerged, particularly pronounced at 50°C, where GVL constituted > 80%–90% of the product distribution for most catalyst systems. This shift in selectivity can be rationalized by considering the reaction mechanism of aqueous phase electrocatalytic LA hydrogenation (Figure 4). The reduction of LA proceeds initially via the hydrogenation of the carbonyl group, yielding 4‐hydroxypentanoic acid (HVA) as an intermediate. Subsequent intramolecular lactonization of HVA forms GVL, a process that involves the nucleophilic attack of the hydroxyl group on the carboxylic acid moiety, with elimination of water to form the five‐membered lactone ring.

The onset temperature for significant GVL formation depended strongly on catalyst formulation and loading. For instance, in the case of CuNi–25 (Figure 3b), GVL selectivity at 5°C remained < 1% across all potentials, while HVA exceeded 83%. However, at 35°C and –2.0 V, GVL selectivity increased sharply to 86.8%. At 50°C, GVL dominated the product spectrum (93.2%–94.2%), with negligible HVA or VA formation. Increasing the metal loading to 50 mC cm^−2^ (Figure 3c) accelerated this transition: at 25°C and –1.6 V, GVL already exceeded 58% compared to only 11% at the lower loading, indicating that higher catalyst surface area promotes both hydrogenation and lactonization by increasing the availability of active sites and potentially extending the residence time of intermediates at the interface.

Incorporating ruthenium into the bimetallic CuNi catalyst significantly enhanced GVL selectivity at all temperatures and potentials. At 15°C and –1.6 V, CuNiRu–25 (Figure 3d) exhibited 26.1% of GVL, whereas CuNi under identical conditions showed only 0.9% GVL. This trend was even more pronounced at higher temperatures, where GVL yields consistently exceeded 90% for all potentials. The synergistic effect of Ru is attributed to its ability to (i) minimize the competing HER, (ii) enhance proton availability, and (iii) provide Lewis acidic sites that stabilize the protonated transition state required for lactonization. Furthermore, at 50 mC cm^−2^ (Figure 3e), the CuNiRu system displayed the highest overall GVL selectivity, reaching 98.5% at –2.0 V and 50°C with no detectable HVA or VA. Notably, even at 15°C, GVL exceeded 26%, outperforming all other systems under the same conditions.

Across all catalyst configurations, the influence of applied potential was most evident at intermediate temperatures. At 35°C, increasing the potential from –1.6 to –2.0 V generally led to a higher GVL yield and a simultaneous reduction in VA, especially on CuNi–25 and CuNiRu–25. This suggests that more negative potentials enhance the surface concentration of reactive hydrogen species, which, in turn, promotes deeper reduction of HVA and/or facilitates faster conversion to GVL when thermally activated lactonization is accessible. At lower temperatures, however, this effect was less pronounced, as lactonization remained the rate‐limiting step.

The formation of VA exhibited a bell‐shaped dependence on temperature. Its selectivity peaked at intermediate temperatures (25°C–35°C), particularly under more cathodic potentials, where HVA accumulated and lactonization was not yet dominant. This trend is consistent with competitive over‐hydrogenation pathways, in which VA is formed via C—O bond hydrogenolysis of HVA or, less likely, directly from LA. As the temperature increased further and cyclization to GVL became more favorable, VA selectivity declined sharply, indicating that efficient lactonization outcompetes over‐hydrogenation routes.

Taken together, these results support a mechanistic proposal (Figure 4) wherein the electrohydrogenation of LA proceeds initially via rapid reduction to HVA, followed by a slower, temperature‐activated lactonization step to form GVL. This latter step appears to be the primary selectivity‐determining event. Catalyst loading enhances surface‐mediated kinetics, while Ru doping significantly lowers the effective activation barrier for lactonization and stabilizes reactive intermediates. The combined effect of temperature, potential, and catalyst architecture thus determines the balance between kinetic trapping at HVA, over‐hydrogenation to VA, and the desired cyclization pathway to GVL [62, 63]. It is important to note that, under the mild aqueous electrocatalytic conditions used here (pH ≈ 0, 5°C–50°C), the formation of VA is most plausibly attributed to over‐reduction of HVA via C—O hydrogenolysis. Extensive literature reports confirm that the five‐membered GVL ring is kinetically and thermodynamically stable in aqueous media, and its ring‐opening requires strong Brønsted acidity and temperatures far above those applied in this study. Thus, direct GVL ring‐opening to VA is not mechanistically supported. The temperature‐dependent behavior observed experimentally—where VA appears only in the regime where transient HVA accumulation is maximal (25°C–35°C)—further reinforces that HVA is the reactive precursor to VA in these systems [64, 65, 66, 67, 68, 69, 70, 71].

These insights provide actionable design principles for tailoring electrochemical systems toward selective biomass upgrading. In particular, operating at temperatures ≥ 35°C, in combination with Ru‐modified electrodes and moderately negative potentials (–1.8 to –2.0 V), enables near‐quantitative conversion of LA to GVL. This underscores the importance of coupling thermal activation with electrochemical bias to overcome kinetic limitations in key transformation steps such as lactonization. Notably, the formation of HVA is favored at lower temperatures (<25°C) where cyclization is suppressed. This suggests that temperature modulation may be strategically employed to direct product selectivity toward either GVL or HVA, depending on the desired valorization route. As such, the integrated approach demonstrated here highlights the versatility of electrocatalytic platforms for tuneable and sustainable conversion of platform molecules such as LA [72, 73, 74].

**FIGURE 5:**
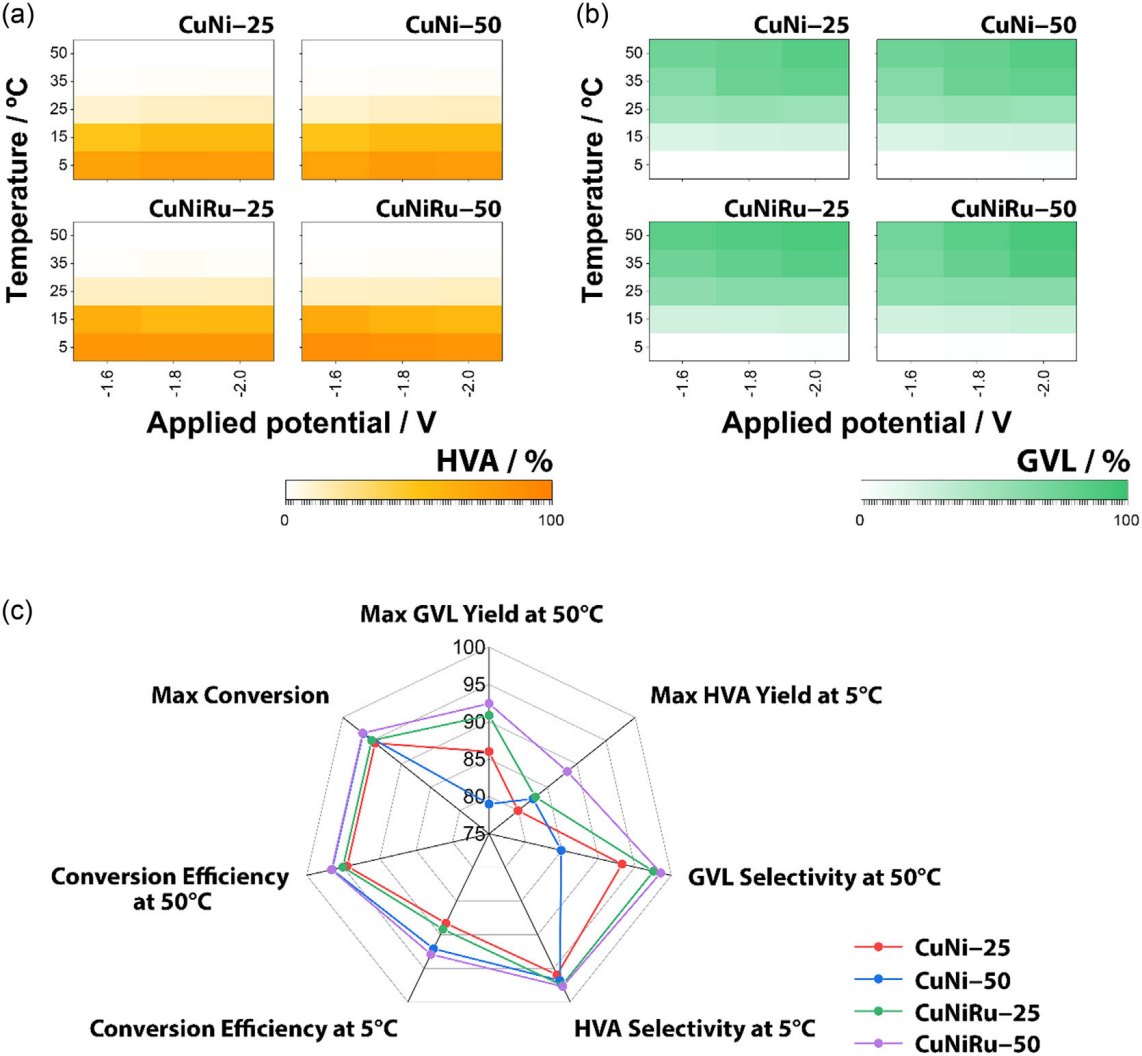
(a) Heat maps showing the yield (%) of 4‐hydroxypentanoic acid (HVA) as a function of applied potential (–1.6 to –2.0 V) and temperature (5°C–50°C) for CuNi and CuNiRu catalysts deposited on GNWs at two different metal loadings: 25 and 50 mC cm^−2^. (b) Corresponding heat maps for GVL yield under identical conditions, illustrating the temperature‐dependent shift from HVA to GVL formation. (c) Comparative radar plot summarizing key catalytic performance metrics for each configuration, including maximum LA conversion, highest HVA and GVL yields, selectivity profiles, and conversion efficiency at low (5°C) and high (50°C) temperatures. All experiments were conducted in triplicate, and average values are presented. These visualizations highlight the combined influence of catalyst composition, loading, temperature, and electrochemical bias on product distribution and efficiency in aqueous‐phase electrocatalytic LA upgrading.

To further elucidate the effect of temperature and applied potential on product distribution, Figure 5a,b presents heat maps of HVA and GVL yields, respectively, across all catalyst configurations. A distinct selectivity shift is observed between 25°C and 35°C, demarcating two kinetically distinct regimes.

At low temperatures (5°C–15°C), HVA formation dominates across all systems, reflecting efficient electrochemical hydrogenation of LA, while the subsequent lactonization to GVL remains thermally limited. Interestingly, the CuNiRu–50 system exhibits exceptionally high HVA yields at 5°C, reaching 88.4%, 86.4%, and 83.4% at –1.6, –1.8, and –2.0 V, respectively. These values indicate that Ru‐containing electrodes effectively promote the LA → HVA step even under subambient conditions, although the ring‐closing lactonization step remains suppressed.

As temperature increases, a sharp decline in HVA selectivity is observed, accompanied by a rise in GVL formation. At 50°C, the CuNiRu–50 electrode achieves GVL yields of 72.6%, 84.5%, and 92.4% at –1.6, –1.8, and –2.0 V, respectively. This dramatic shift reflects the overcoming of the lactonization barrier, especially under more cathodic bias, and confirms the synergistic role of Ru and thermal activation in promoting the final GVL‐forming steps. These results underscore the tunability of the system: at low temperatures, CuNiRu–50 favors selective accumulation of HVA, while at elevated temperatures and negative potentials, it enables nearly quantitative conversion to GVL.

In addition, ^1^H NMR analyses were conducted to confirm the formation of HVA at low temperatures (5°C) and GVL at mild temperatures (50°C). As shown in Figure S6, HVA is the predominant product at 5°C, while Figure S7 illustrates that GVL is the main product at 50°C. Additionally, the purification of GVL from aqueous reaction mixtures containing LA, HVA, and VA was achieved through sequential liquid–liquid extraction and pH‐controlled partitioning. This approach exploits the differences in acidity (p*K*_a_) and functional group chemistry between carboxylic acids and the lactone. LA (p*K*_a_ ≈ 4.6), VA (p*K*_a_ ≈ 4.8), and HVA (expected p*K*_a_ < 4.8 due to the hydroxyl substituent) predominantly exist in their neutral, protonated forms under acidic conditions (pH < 3), making them moderately soluble in organic solvents such as ethyl acetate. Upon treatment with aqueous sodium bicarbonate (NaHCO_3_, pH ≈ 8.4), these acids are deprotonated to form their corresponding carboxylate anions, which are highly water‐soluble and thus extracted into the aqueous phase. In contrast, GVL—being a neutral lactone without a carboxylic acid group—remains uncharged and nonpolar under these conditions, and thus persists in the organic phase throughout the washing steps. This differential solubility enables its selective isolation and effective purification through acid–base extraction.

Finally, to support the temperature–potential maps and provide a comprehensive overview of catalyst performance, Figure 5c presents a comparative radar plot incorporating seven key metrics: maximum GVL yield at 50°C, maximum HVA yield at 5°C, GVL selectivity at 50°C, HVA selectivity at 5°C, conversion efficiency at both 5°C and 50°C, and overall maximum conversion. The data, obtained from triplicate experiments and averaged, offer a multidimensional evaluation of each catalyst configuration.

Among the four systems, CuNiRu–50 displays the most balanced and extensive profile, outperforming all others in nearly every category. It achieves the highest GVL yield at 50°C (92.4%), the greatest GVL selectivity (98.5%), and excellent conversion efficiency (96.6%). These results confirm the synergistic effect of Ru incorporation and increased catalyst loading, which together enable fast hydrogenation and efficient thermally driven lactonization. In contrast, CuNi–25 shows the most limited performance, with comparatively lower GVL yield (86.0%) and selectivity (93.2%) at high temperature, despite acceptable conversion efficiency. This underscores the limitations imposed by low metal loading and the absence of Ru, both of which constrain the surface availability of active sites and the catalyst's ability to overcome kinetic barriers associated with ring‐closure.

CuNi–50 and CuNiRu–25 occupy intermediate positions on the radar plot, highlighting the distinct roles of loading and composition. CuNi–50 mC benefits from increased surface area (leading to high conversion). Conversely, CuNiRu–25 exhibits excellent selectivity (97.5% GVL at 50°C) but slightly reduced yield, suggesting that neither factor alone is sufficient to fully optimize the system. Only the CuNiRu–50 configuration achieves simultaneous maximization of conversion, selectivity, and yield, resulting in a near‐ideal, symmetrical radar profile.

Two broader trends emerge from this analysis. First, HVA selectivity at low temperature (>95% at 5°C) is consistent across all materials, confirming that the LA‐to‐HVA hydrogenation step is rapid and not significantly influenced by catalyst composition. Second, GVL formation at elevated temperature is strongly catalyst‐dependent, reinforcing that lactonization is the thermally activated, rate‐determining step, which can be facilitated by both surface area and Ru‐induced electronic or acid–base effects. In summary, the radar plot provides a holistic visualization of performance, confirming that optimal electrocatalytic upgrading of LA to GVL requires both structural (high roughness) and compositional (Ru presence) optimization. These findings highlight the value of integrated catalyst design strategies and point to CuNiRu–50 as a promising benchmark system for efficient and tuneable biomass valorization.

To complement the electrocatalytic performance data, the architectural durability of CuNi and CuNiRu electrodes was assessed by quantifying metal leaching after electrolysis in 0.5 M LA solutions at different temperatures and applied potentials. The fraction of metal lost from the electrode surface after a single‐electrolysis cycle was determined by ICP‐OES and is summarized in Table S2. CuNi electrodes showed significant susceptibility to degradation under all conditions tested. At 25°C, the total leached fraction increased from 11.4% at –1.6 V to 26.4% at –2.0 V. This clear potential‐dependent rise indicates that more reductive environments promote dissolution of surface‐exposed Cu and Ni sites, likely via the reductive stripping of surface oxides or hydroxides and LA chelation of metal cations. The effect of temperature was even more pronounced: at –2.0 V, increasing the temperature from 5°C to 50°C led to a > 3.4‐fold increase in metal loss (from 11.3% to 38.2%). These findings confirm that CuNi is highly vulnerable to both electrochemical and thermal stress, which could severely limit its operational lifetime. In stark contrast, CuNiRu electrodes displayed exceptional stability. Metal leaching remained undetectable (below 0.1%) at low‐to‐moderate temperatures and potentials (≤25°C, ≤–1.8 V) and rose only marginally at more aggressive conditions. At the highest temperature and bias tested (50°C, –2.0 V), the metal loss was just 1.1%—over 35 times lower than that of the corresponding CuNi electrode. Moreover, Ru leaching was consistently below the detection limit under all tested conditions, indicating that the noble metal is stably integrated within the catalyst structure and does not undergo reductive or oxidative dissolution during operation. The superior stability of CuNiRu can be rationalized by several factors. First, the presence of Ru forms a more noble, passivating surface layer that protects the underlying CuNi matrix. Second, Ru's high intrinsic HER activity facilitates rapid *H generation and proton consumption, limiting local alkalinization and the formation of soluble metal hydroxide complexes. Third, galvanic coupling between Ru and the base metals may shift the local redox potential, kinetically hindering the reductive dissolution of Cu and Ni. These mechanisms collectively suppress the electrochemical pathways responsible for catalyst degradation in bimetallic systems. In addition, postelectrolysis FE‐SEM micrographs reveal that the CuNiRu catalyst retains its structural architecture and surface morphology, indicating excellent stability under operating conditions.

To complement the single‐cycle leaching analysis and directly probe operational robustness, the reusability of the CuNiRu–50 catalyst was examined through five consecutive chronoamperometric runs at 50°C in 0.5 M LA (15 mL, 1500 C per run) using the same electrode. The CuNiRu–50 electrode displayed highly reproducible performance throughout the sequence. LA conversion, FE, and product distribution remained essentially constant from the first to the fifth cycle, with variations below 6% in all cases. GVL systematically remained the dominant product, and no progressive increase of side products was detected (Table S3). Importantly, ICP‐OES analysis of the electrolytes collected after each cycle revealed metal leaching values comparable to those obtained in the single‐cycle experiments discussed above, with no systematic increase along the cycling sequence. This confirms that no significant cumulative dissolution of Cu, Ni, or Ru occurs under repeated operation. The negligible leaching is consistent with the preserved nanostructured morphology observed by postelectrolysis FE‐SEM and further supports the structural robustness of the CuNiRu–GNW architecture. The response of CuNiRu–50 under more concentrated conditions was evaluated in 4.0 M LA at 50°C (15 mL, 12 000 C). Despite the substantially increased substrate loading, the catalyst preserved the same qualitative selectivity pattern, with GVL as the main product. Compared to the 0.5 M benchmark, the overall FE decreased by approximately 8%. This moderate penalty is attributed to the altered physicochemical properties of the highly concentrated electrolyte (e.g., increased viscosity, modified conductivity and interfacial environment), which may favor a slightly higher contribution of nonproductive cathodic pathways, including hydrogen evolution, under the same nominal potential. Importantly, the product distribution remains dominated by GVL, with a slightly higher GVL fraction in the liquid products at 4.0 M LA, and metal leaching remains low, indicating that concentrated operation does not compromise catalyst integrity or selectivity. Consistently, ICP‐OES analysis under these conditions confirmed metal dissolution levels in the same low range as for 0.5 M LA, indicating that the increased LA concentration does not compromise the integrity of the trimetallic layer. Overall, these results demonstrate that (i) the CuNiRu–50 catalyst sustains stable activity and selectivity over multiple cycles, (ii) metal leaching remains minimal and noncumulative, and (iii) the catalyst retains its temperature‐controlled selectivity toward GVL even at high LA concentrations, reinforcing its relevance for practical electrocatalytic upgrading processes.

Beyond product selectivity and conversion, the overall performance of the electrochemical hydrogenation process must also be evaluated in terms of energy efficiency and storage viability. To this end, three complementary metrics were examined: Faradaic efficiency (FE); energy consumption (EC); and energy storage efficiency (ESE). Together, these parameters provide a comprehensive understanding of the trade‐offs between catalytic activity, selectivity, and energy conversion under varying electrochemical and thermal conditions [75].

The FE quantifies the fraction of the total charge passed that is effectively utilized in the electrochemical hydrogenation of LA to liquid‐phase products. As shown in Tables S4 and S5, FE strongly depends on the applied potential and reaction temperature. At moderate cathodic potentials (–1.6 V), the FE values are maximized across all catalyst formulations. At higher overpotentials (–1.8 V and –2.0 V), a consistent decrease in FE is observed due to the competitive HER, which consumes a greater fraction of the input electrons. The decline in FE is particularly notable at –2.0 V, where values drop by 10%–20% depending on the catalyst and temperature. Increasing the temperature generally enhances FE up to ~ 35°C due to improved reaction kinetics and mass transport. However, at 50°C, a slight decrease in FE is again observed, suggesting increased HER activity and proton mobility offset the kinetic advantages. Catalysts with higher metal loading (50 mC cm^−2^) consistently outperformed their 25 mC cm^−2^ counterparts in FE, attributable to enhanced active site density and more efficient charge distribution. The incorporation of Ru appears to reduce FE slightly (by ~ 4%–6%) due to its higher *H adsorption strength, which likely enhances HER over LA reduction.

The EC, calculated in kWh per mole of product, reflects the total electrical energy required to generate the liquid‐phase hydrogenation products. As expected, EC shows an inverse relationship with FE and is lowest under conditions that favor efficient charge utilization and moderate current densities. At –1.6 V, EC values for all catalyst systems fall within **0**.121–0.127  kWh mol^−1^, increasing progressively to 0.126–0.133  kWh mol^−1^ at –1.8 V and reaching 0.130–0.142  kWh mol^−1^ at –2.0 V. Temperature also plays a favorable role, with EC slightly decreasing at higher temperatures (up to 35°C–40 °C), likely due to improved electrolyte conductivity and decreased ohmic resistance. The lowest EC (0.121  kWh mol^−1^) was recorded for CuNiRu–50 at –1.6 V and 50 °C, representing an energy‐efficient regime with limited HER and high conversion. Comparatively, CuNi–50 demonstrated slightly lower EC at similar conditions, suggesting a better balance between LA selectivity and HER. The increase in EC at more cathodic potentials is modest but consistent, supporting the conclusion that deeper reduction potentials introduce diminishing returns with respect to energy efficiency [75].

ESE captures the fraction of input electrical energy stored in the chemical bonds of stable liquid products (GVL, HVA, VA). This metric integrates both FE and the combustion enthalpy gain associated with the hydrogenation of LA into each product. Given the higher energy density of VA (+0.42 MJ mol^−1^), ESE is enhanced under conditions that selectively favor its formation without excessive HER losses. The highest ESE value, 71.5%, was achieved with CuNi–50 at –1.6 V and 35 °C (FE =  89.2%, VA =  44.7%), indicating a near‐optimal balance between selectivity, conversion, and current efficiency. In contrast, CuNiRu‐50 reached a slightly lower maximum of 64.4% under the same conditions due to its lower FE and reduced VA selectivity. The ESE values progressively decrease at –2.0 V due to lower FE and excessive HER. Conversely, at low temperatures (5°C–15°C), although FE may remain relatively high, ESE values drop below 50% due to a dominant formation of HVA, which has a lower combustion enthalpy. Overall, the combination of moderate potential (–1.6 V), elevated temperature (35 °C), and a high‐density Ni‐based catalyst enables the most favorable conditions for maximizing both energy efficiency and storage density. These findings underscore the importance of product distribution control and HER suppression in optimizing electrochemical hydrogenation systems for renewable electricity storage [75].

The CuNi‐ and CuNiRu‐based catalysts demonstrate comparable or superior FE, EC, and ESE metrics when benchmarked against previously reported Pb‐based systems operating under acidic conditions [13, 14, 75, 76]. For clarity and completeness, a quantitative comparison with representative Pb‐, Cd‐, In‐, Ni‐, and Pt‐based electrocatalytic systems, including conversion, selectivity, FE, EC, and stability (when available), is provided in Table S6. This comparison highlights that the CuNiRu/GNW catalyst matches or exceeds the performance of most state‐of‐the‐art Pb systems while avoiding the environmental and toxicity concerns inherent to lead‐based cathodes. Notably, CuNi–50 achieves similar or better ESE values (~71%) without the environmental drawbacks associated with lead electrodes. The codeposition of Ru marginally enhances LA conversion and low‐temperature performance but introduces a small trade‐off in FE due to increased HER. This study highlights the critical role of tuning the electron‐to‐product coupling, hydrogen binding properties, and reaction energetics to maximize ESE in biomass‐derived liquid fuel synthesis. CuNi/GNW platforms, especially at optimized deposition densities and moderate overpotentials, represent a promising and sustainable route for converting surplus renewable electricity into energy‐dense, storable biofuels.

## 4 Conclusions

This work demonstrates that coupling earth‐abundant CuNi electrocatalysts with small amounts of ruthenium (Ru) on vertically aligned GNWs offers a sustainable, energy‐efficient, and atom‐economical strategy for the electrocatalytic upgrading of LA in aqueous media. The unique architecture of the GNW scaffold—featuring high surface area, excellent electrical conductivity, and structural robustness—supports the uniform dispersion and stable anchoring of the multimetallic coating. Within a broad operational temperature range (5°C–50°C) and under three applied negative potentials (–1.6 to –2.0 V), all four investigated configurations (CuNi–25, CuNi–50, CuNiRu–25, and CuNiRu–50) demonstrated rapid LA conversion exceeding 82% while simultaneously suppressing the formation of gaseous or volatile side‐products.

Mechanistic analysis based on product distribution and NMR confirmed that HVA was consistently the primary intermediate across all systems. At 5°C, the selectivity toward HVA surpassed 95% for each catalyst, indicating that the initial hydrogenation of the carbonyl group in LA is a fast and largely catalyst‐insensitive process. However, subsequent lactonization of HVA to GVL emerged as the thermally activated, rate‐determining step, strongly influenced by both catalyst composition and morphology. This transformation required higher temperature to proceed efficiently, particularly above 35°C. These findings confirm that temperature modulation is not only a practical tool for directing the LA → GVL/HVA pathway but also a broadly applicable strategy for controlling selectivity in other multistep electrocatalytic biomass‐upgrading reactions.

Notably, increasing the deposited metal loading from 25 to 50 mC cm^−2^ enlarged the electroactive surface area, improving conversion but only marginally affecting GVL selectivity. In contrast, the strategic incorporation of a small amount of Ru yielded a remarkable enhancement in selectivity and yield. Ru appears to play a pivotal role in modulating electronic effects, facilitating *H spillover, stabilizing reaction intermediates, and suppressing competing hydrogen evolution. These synergistic effects culminated in the CuNiRu–50 configuration, which achieved 96.6% conversion, 92.4% GVL yield, and 98.5% selectivity at 50°C, with minimal catalyst metal leaching (<1%). This system exhibited a near‐ideal radar performance profile and can be considered a benchmark architecture for efficient, selective, and scalable biomass valorization.

The temperature–potential maps developed in this study serve as practical tools for tuning product selectivity. Operating at low temperature (<15°C) favors HVA production, a valuable precursor for bioplastics and polyesters. At moderate temperatures (≈50°C), GVL formation is maximized, providing an attractive platform molecule for green solvents, fuel additives, and fine chemicals. Importantly, the reaction proceeds under ambient pressure and in aqueous medium, with the electrolyte itself serving as a hydrogen donor, and without the need for external H_2_. This significantly reduces the energy input, safety risks, and carbon footprint compared to conventional thermocatalytic hydrogenation processes.

In addition to high conversion and tuneable selectivity, the system demonstrated outstanding Faradaic efficiency (up to 89%), low energy consumption (as low as 0.121 kWh mol^−1^), and energy storage efficiencies approaching 72%, highlighting its potential for direct, electricity‐to‐liquid fuel conversion from renewable sources. The combination of high Faradaic efficiency, low specific energy demand, and selective product control positions the CuNiRu–50/GNW system as a reference architecture for energy‐efficient electrocatalytic hydrogenation in aqueous media.

Beyond performance, this study highlights the broader significance of temperature modulation as a powerful yet underutilized parameter in electrocatalysis. By tuning the thermal environment in tandem with catalyst composition and structure, the product distribution and efficiency of bioupgrading reactions can be precisely controlled. Furthermore, the GNW support proves ideal for dispersing and stabilizing multimetallic thin films, offering mechanical integrity, chemical stability, and scalability. Looking ahead, future research should explore integration of these electrocatalytic systems into renewable‐powered flow reactors for continuous operation, while extending the catalytic platform toward downstream GVL transformation into higher value chemicals. Such efforts will contribute to the development of decentralized, carbon‐neutral, and circular biorefinery concepts aligned with global sustainability targets. Overall, this study establishes a tuneable, low‐pressure, water‐compatible electrocatalytic platform that offers unprecedented control over product distribution, setting the basis for scalable, modular, and renewable‐powered biorefinery schemes.

## Supporting Information

Additional supporting information can be found online in the Supporting Information Section. **Supporting Fig. S1**: Cyclic voltammograms recorded at 25°C on a graphene nanowalls (GNWs) electrode in (a) CuNi and (b) CuNiRu electrolytes, using a scan rate of 50 mV s^−^¹. Measurements were performed in concentrated electrochemical baths prepared according to the compositions detailed in Table S1. **Supporting Fig. S2**: Field‐emission scanning electron microscopy (FE‐SEM) micrographs of pristine graphene nanowalls (GNWs). Scale bar: 200 nm. **Supporting Fig. S3**: Field‐emission scanning electron microscopy (FE‐SEM) micrographs of (a) CuNi‐ and (b) CuNiRu‐decorated graphene nanowalls (GNWs). Deposits were prepared potentiostatically at –1.0 V vs. Ag|AgCl, with a deposition charge density of 25 mC cm^−2^. Scale bar: 200 nm. **Supporting Fig. S4**: X‐ray diffraction (XRD) patterns of graphene nanowalls (GNWs) decorated with CuNi and CuNiRu deposits. **Supporting Fig. S5**: High‐resolution XPS spectra of GNW‐supported deposits: (a) Cu 2p, (b) Ni 2p, (c) O 1s, and (d) Ru 3p for graphite nanowalls (GNWs) decorated with CuNi and CuNiRu coatings. **Supporting Fig. S6**: ^1^H NMR spectrum (400 MHz, CDCl3) of a reaction mixture containing HVA (blue, left) and GVL (green, right). The spectrum displays characteristic resonances for both compounds, with blue dots indicating the proton signals associated to HVA and green dots corresponding to GVL. The sample was obtained from CuNiRu‐25 electrolysis at 5°C. The overlapping signals confirm the presence of both species in the mixture. **Supporting Fig. S7**:^1^H NMR spectra (400 MHz, CDCl3) of: (a) the crude reaction mixture obtained after CuNiRu‐catalyzed electrolysis (25 Mc cm^‐2^) at 50°C, showing predominantly GVL (green) along with minor signals from HVA (blue); (b) the organic phase isolated after sequential liquid–liquid extraction of the reaction mixture with ethyl acetate and aqueous sodium bicarbonate (5%). The resulting spectrum corresponds to pure GVL obtained, confirming successful separation of HVA and the high selectivity of the process. **Supporting Table S1**: Composition of concentrated and diluted electrochemical baths used for the electrodeposition of CuNi and CuNiRu‐based coatings. **Supporting Table S2**: Fraction of total metal leached from CuNi and CuNiRu electrodes after one electrolysis cycle, as determined by ICP‐OES, under different applied potentials and temperatures in 0.5 M levulinic acid. “ND” indicates leaching below the detection limit. **Supporting Table S3**: Summary of the electrocatalytic reusability tests (five consecutive cycles) and the high‐concentration LA experiment performed with the CuNiRu/GNW catalyst. Experimental conditions and resulting product selectivities (GVL, VA, HVA) and overall LA conversion are reported for each run. **Supporting Table S4**: Electrocatalytic performance of CuNi‐based catalysts (CuNi‐25 and CuNi‐50) for the electrochemical hydrogenation of levulinic acid (LA) at different temperatures (5–50°C) and applied potentials (–1.6, –1.8, and –2.0 V vs. Ag|AgCl). For each condition, LA conversion (%), product distribution (GVL, HVA, VA, and other products (OP), all in mol%), Faradaic efficiency (FE, %), energy consumption (EC, in kWh mol^−1^), and energy storage efficiency (ESE, %) are reported. CuNi‐25 and CuNi‐50 refer to catalysts electrodeposited at 25 and 50 mC cm^−2^, respectively. GVL: γ‐valerolactone; HVA: 4‐hydroxypentanoic acid; VA: valeric acid. **Supporting Table S5**: Electrocatalytic performance of CuNiRu‐based catalysts (CuNi‐25 and CuNi‐50) for the electrochemical hydrogenation of levulinic acid (LA) at different temperatures (5–50 °C) and applied potentials (–1.6, –1.8, and –2.0 V vs. Ag|AgCl). For each condition, LA conversion (%), product distribution (GVL, HVA, VA, and other products (OP), all in mol%), Faradaic efficiency (FE, %), energy consumption (EC, in kWh mol^−^¹), and energy storage efficiency (ESE, %) are reported. CuNi‐25 and CuNi‐50 refer to catalysts electrodeposited at 25 and 50 mC cm^−2^, respectively. GVL: γ‐valerolactone; HVA: 4‐hydroxypentanoic acid; VA: valeric acid. **Supporting Table S6**: Comparison of CuNiRu/GNW performance with representative Pb‐, Cd‐, In‐, Ni‐, Pt‐ and carbon‐based electrocatalysts for LA reduction, including conversion, main product, selectivity and FE.

## Funding

This study was supported by Agència de Gestió d'Ajuts Universitaris i de Recerca (Grand 2023 CLIMA 00009).

## Conflicts of Interest

The authors declare no conflicts of interest.

## Supporting information

Supplementary Material

## Data Availability

The data that support the findings of this study are available from the corresponding author upon reasonable request.
