# Supplementary material for: Temperature‐Tuned Electrocatalytic Valorization of Levulinic Acid to γ‐Valerolactone or 4‐Hydroxyvaleric Acid over CuNi(Ru)/Graphene Nanowalls
Source: ChemSusChem. 2025 Nov 25;19(1):e202502403. doi: 10.1002/cssc.202502403 (PMC12767560; doi:10.1002/cssc.202502403)
Supplement: Supplementary file 1 — Supplementary Material [file CSSC-19-e202502403-s001.pdf]

Supporting Information

Temperature-Controlled Sustainable Electrocatalytic Valorisation of Levulinic Acid to γ-Valerolactone or 4-Hydroxyvaleric Acid Using CuNi (Ru)/Graphene Nanowalls

Pol Vilariño[1,2], Jordi Rigual-Miret[1,2], Ghulam Farid [2,3], Stefanos Chaitoglou [2,3], Roger Amade [2,3], Elvira Gómez [1,2,*] and Albert Serrà [1,2,*]

[1] Grup d'Electrodeposició de Capes Primes i Nanoestructures (GE-CPN), Departament de Ciència de Materials i Química Física, Universitat de Barcelona, Martí i Franquès, 1, E-08028, Barcelona, Catalonia, Spain.

[2] Institute of Nanoscience and Nanotechnology (IN^{2}UB), Universitat de Barcelona, Barcelona, Catalonia, Spain.

[3] Department of Applied Physics, Universitat de Barcelona, Martí i Franquès, 1, E-08028, Barcelona, Catalonia, Spain.

Corresponding author: a.serra@ub.edu (A.S.)

Table of contents

CuNi and CuNiRu Electrodeposition Baths

- Table S1: Composition of concentrated and diluted electrochemical baths for CuNi and CuNiRu coatings

Cyclic Voltammetry of CuNi and CuNiRu Baths on Graphene Nanowalls (GNWs)

- Figure S1: Cyclic voltammograms recorded at 25 °C on GNWs in CuNi and CuNiRu electrolytes

FE-SEM Micrographs of Pristine GNWs

- Figure S2: Morphology of pristine GNWs (200 nm scale)

FE-SEM Micrographs of GNWs Decorated with CuNi and CuNiRu Deposits

- Figure S3: Morphology of CuNi- and CuNiRu-decorated GNWs

XRD Patterns of GNWs Decorated with CuNi and CuNiRu Deposits

- Figure S4: X-ray diffraction patterns of GNW-supported CuNi and CuNiRu coatings

XPS Spectra of GNWs Decorated with CuNi and CuNiRu Deposits

- Figure S5: High-resolution Cu 2p, Ni 2p, O 1s, and Ru 3p spectra

NMR Spectra of Obtained Products

- Figure S6: 1H NMR of mixture with HVA and GVL (CuNiRu-25 at 5 °C)
- Figure S7: 1H NMR of crude and purified products at 50 °C (CuNiRu-25)

Catalyst Stability and Metal Leaching under Electrolysis Conditions

- Table S2: Metal leaching from CuNi and CuNiRu electrodes (ICP-OES)
- Table S3: Reusability and high-concentration electrocatalysis results for the CuNiRu/GNW catalyst.

Efficiency Parameters

- Equations for Faradaic efficiency (FE), energy consumption (EC), and energy storage efficiency (ESE)
- Table S4: Conversion, selectivity, FE, EC, and ESE of CuNi-25 and CuNi-50 at various conditions

- Table S5: Conversion, selectivity, FE, EC, and ESE of CuNiRu-25 and CuNiRu-50 at various conditions
- Table S6: Comparison of CuNiRu/GNW performance with representative Pb-, Cd-, In-, Ni-, Pt- and carbon-based electrocatalysts for LA reduction.

CuNi and CuNiRu Electrodeposition Baths

Table S1: Composition of concentrated and diluted electrochemical baths used for the electrodeposition of CuNi and CuNiRu-based coatings.

	CuNi bath		CuNiRu bath	
	Concentrated bath	Diluted bath	Concentrated bath	Diluted bath
$NiCl_2$/M	0.30	0.030	0.30	0.030
$CuCl_2$/M	0.05	0.005	0.05	0.005
$RuCl_3$/M	0.00	0.00	0.02	0.002
$C_6H_5Na_3O_7$/M	0.20	0.200	0.20	0.200
NaCl/M	0.05	0.005	0.00	0.000

Cyclic Voltammetry of CuNi and CuNiRu Baths on Graphene Nanowalls (GNWs)

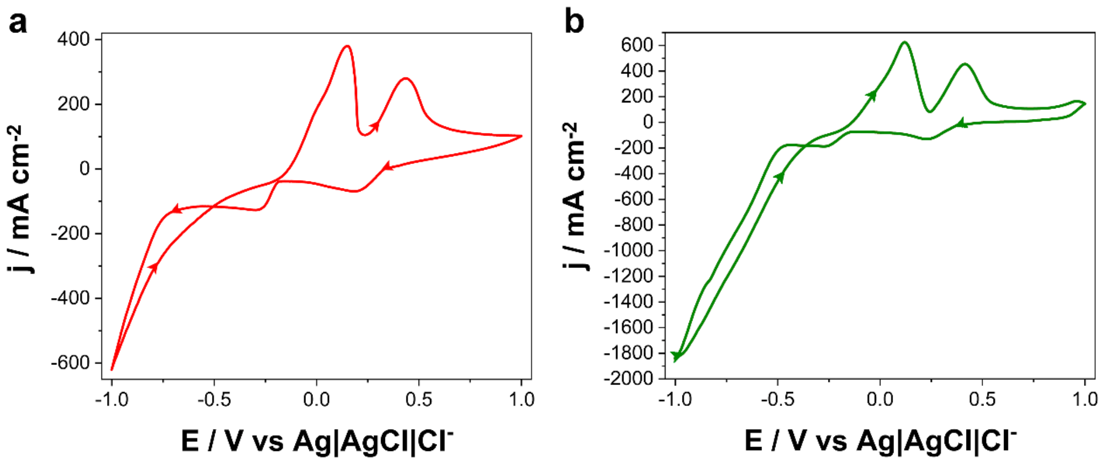

Figure S1: Cyclic voltammograms recorded at 25 °C on a graphene nanowalls (GNWs) electrode in (a) CuNi and (b) CuNiRu electrolytes, using a scan rate of 50 mV s^{-1}. Measurements were performed in concentrated electrochemical baths prepared according to the compositions detailed in Table S1.

FE-SEM Micrographs of Pristine Graphene Nanowalls (GNWs)

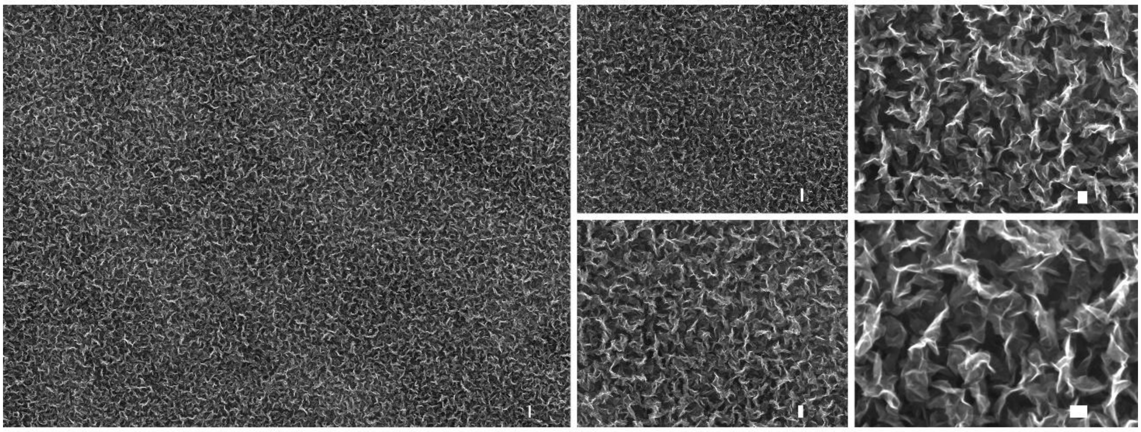

Figure S2. Field-emission scanning electron microscopy (FE-SEM) micrographs of pristine graphene nanowalls (GNWs). Scale bar: 200 nm.

FE-SEM Micrographs of Graphene Nanowalls (GNWs) Decorated with CuNi and CuNiRu Deposits

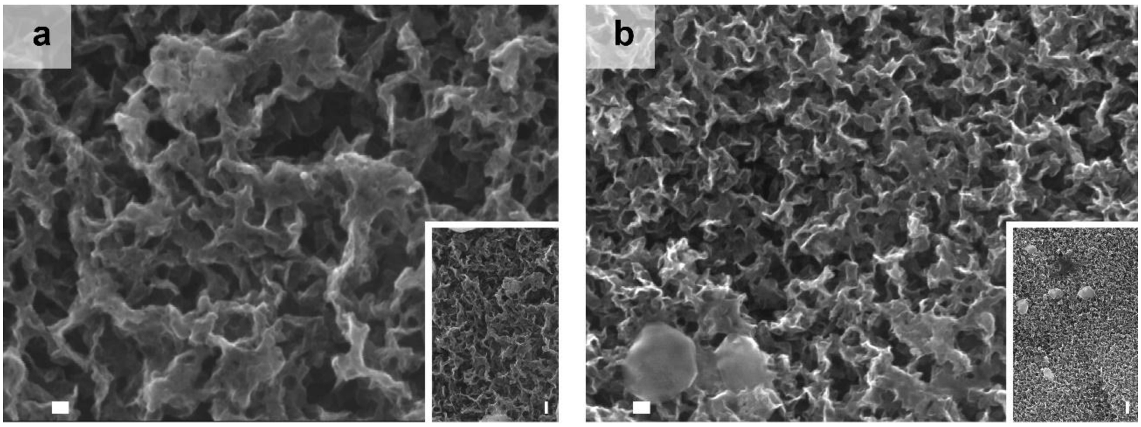

Figure S3. Field-emission scanning electron microscopy (FE-SEM) micrographs of (a) CuNi- and (b) CuNiRu-decorated graphene nanowalls (GNWs). Deposits were prepared potentiostatically at –1.0 V vs. Ag|AgCl, with a deposition charge density of 25 mC cm^{-2}. Scale bar: 200 nm.

XRD patterns of Graphene Nanowalls (GNWs) Decorated with CuNi and CuNiRu Deposits

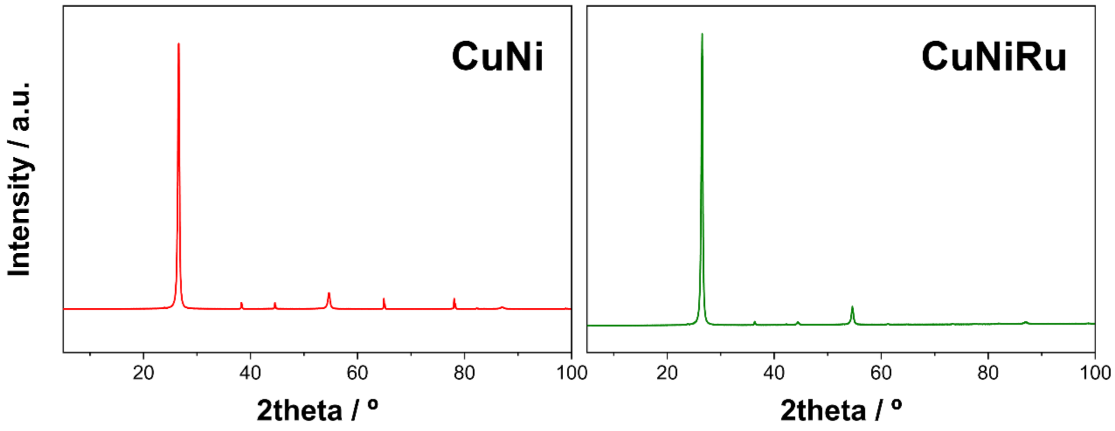

Figure S4: X-ray diffraction (XRD) patterns of graphene nanowalls (GNWs) decorated with CuNi and CuNiRu deposits.

XPS spectra of Graphene Nanowalls (GNWs) Decorated with CuNi and CuNiRu Deposits

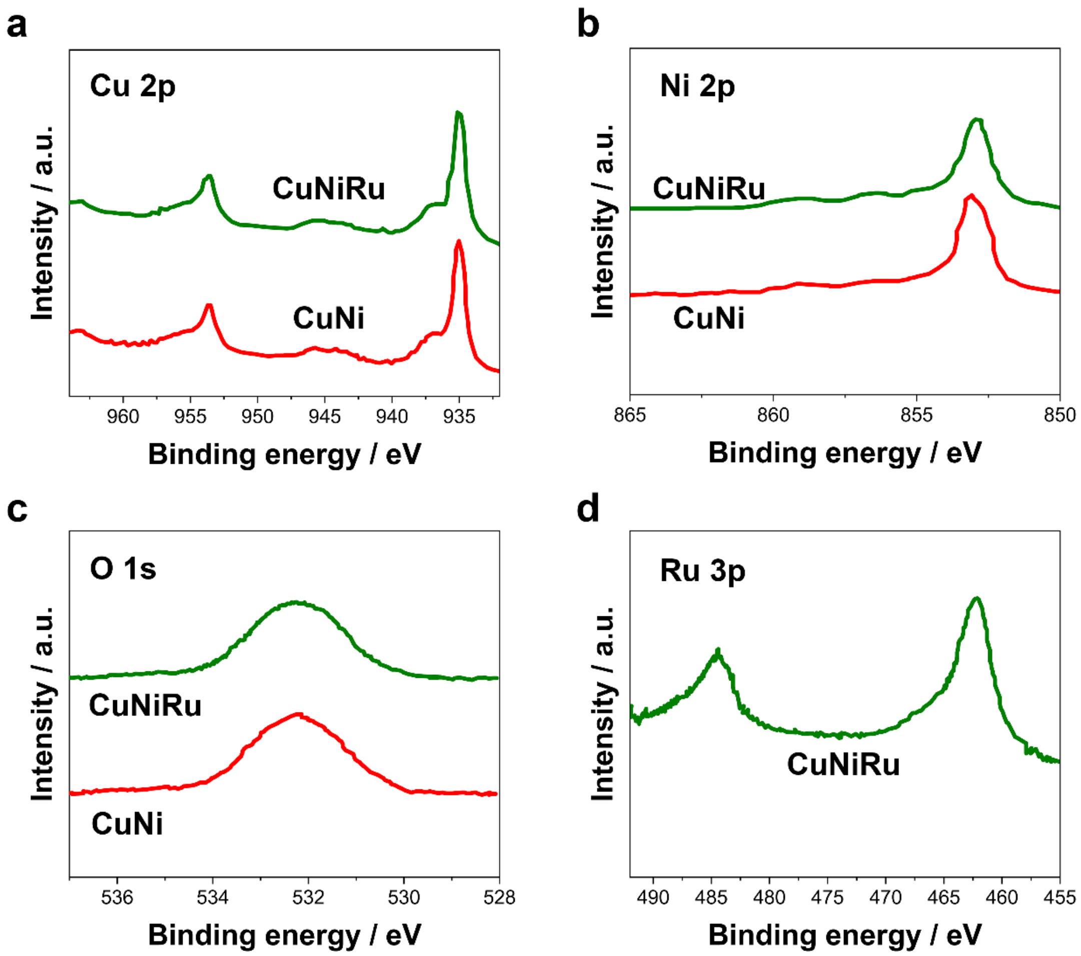

Figure S5: High-resolution XPS spectra of GNW-supported deposits: (a) Cu 2p, (b) Ni 2p, (c) O 1s, and (d) Ru 3p for graphite nanowalls (GNWs) decorated with CuNi and CuNiRu coatings.

NMR spectra of obtained products

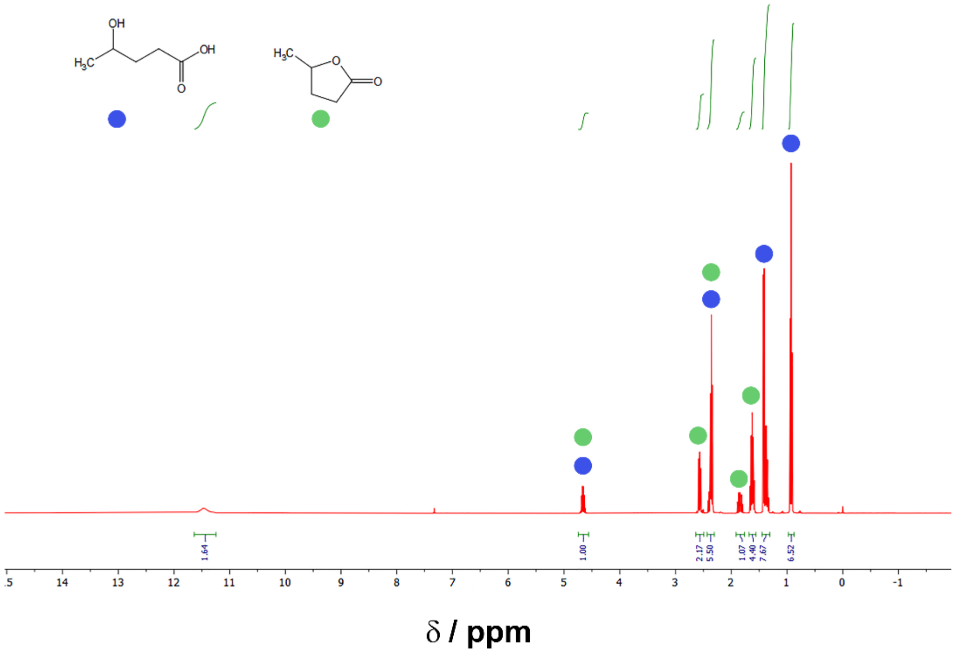

Figure S6: ^{1}H NMR spectrum (400 MHz, $CDCl_3$) of a reaction mixture containing HVA (blue, left) and GVL (green, right). The spectrum displays characteristic resonances for both compounds, with blue dots indicating the proton signals associated to HVA and green dots corresponding to GVL. The sample was obtained from CuNiRu-25 electrolysis at 5 °C. The overlapping signals confirm the presence of both species in the mixture.

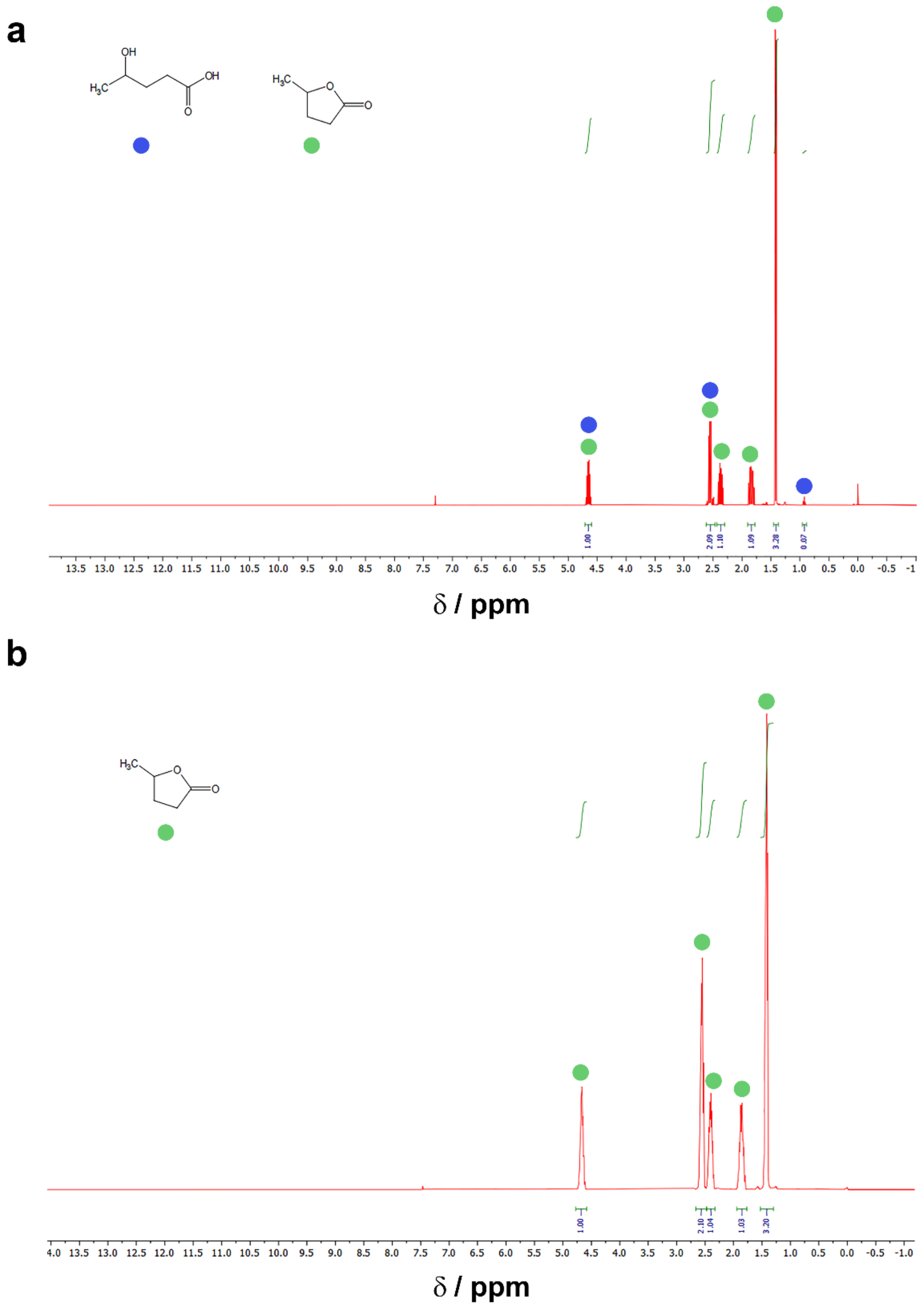

Figure S7: ^{1}H NMR spectra (400 MHz, $CDCl_3$) of: (a) the crude reaction mixture obtained after CuNiRu-catalyzed electrolysis (25 Mc cm^{-2}) at 50 °C, showing predominantly GVL (green) along with minor signals from HVA (blue); (b) the organic phase isolated after sequential liquid–liquid extraction of the reaction mixture with ethyl acetate and aqueous sodium bicarbonate (5 %). The resulting spectrum corresponds to pure GVL obtained, confirming successful separation of HVA and the high selectivity of the process.

Catalyst Stability and Metal Leaching Behavior under Electrolysis Conditions

Table S2: Fraction of total metal leached from CuNi and CuNiRu electrodes after one electrolysis cycle, as determined by ICP-OES, under different applied potentials and temperatures in 0.5 M levulinic acid. "ND" indicates leaching below the detection limit.

Temperature (°C)	CuNi −1.6 V	CuNi −1.8 V	CuNi −2.0 V	CuNiRu −1.6 V	CuNiRu −1.8 V	CuNiRu −2.0 V
5	6.20%	9.20%	11.30%	ND	ND	ND
15	8.30%	13.60%	16.90%	ND	ND	0.20%
25	11.40%	17.90%	26.40%	ND	0.40%	0.50%
35	14.60%	24.30%	33.20%	0.40%	0.80%	0.90%
50	21.90%	29.80%	38.20%	0.60%	1.00%	1.10%

Table S3: Summary of the electrocatalytic reusability tests (five consecutive cycles) and the high-concentration LA experiment performed with the CuNiRu/GNW catalyst. Experimental conditions and resulting product selectivities (GVL, VA, HVA) and overall LA conversion are reported for each run.

Experiment	[LA] / M	Volume / mL	E / V	Circulated charge / C	GVL / %	VA / %	HVA / %	Conversion / %
Reusability 1	0.5	15	-2.0	1500	98.1	1.6	0.0	92.4
Reusability 2	0.5	15	-2.0	1500	98.3	1.4	0.0	91.5
Reusability 3	0.5	15	-2.0	1500	97.9	1.8	0.0	90.1
Reusability 4	0.5	15	-2.0	1500	97.6	2.1	0.0	89.4
Reusability 5	0.5	15	-2.0	1500	97.4	2.3	0.0	89.9
High concentration	4.0	15	-2.0	12000	99.3	0.5	0.0	86.4

Efficiency parameters

The Faradaic efficiency (FE) of the electrochemical hydrogenation (ECH) of levulinic acid (LA) was calculated according to the following equation:

$$\boldsymbol{FE}(\%) = \sum\left(\frac{Mole\ of\ produced\ product\ i\ \times\ n_i\ \times\ F}{Total\ charge\ passed}\right) \times 100$$

where $\boldsymbol{n_i}$ is the number of electrons required for the formation of the respective product (with n=2 for γ-valerolactone (GVL) and 4-hydroxyvaleric acid (HVA), and n=4 for valeric acid (VA) – other minority products were not considered), F is the Faraday constant (96485 C mol^{-1}), and total charge passed is the total charge passed during electrolysis (in coulombs).

To evaluate the energy efficiency of the ECH process, the energy consumption (EC) was determined as:

$$\boldsymbol{EC}\ \left(\boldsymbol{kWh\ mol_{product}^{-1}}\right) = \frac{electrical\ energy\ input}{product\ unit} = \sum\left(\frac{E\ \times\ I\ \times\ t/\ 1000}{mole\ of\ product\ i}\right)$$

where E is the applied potential (V vs. RHE), I is the current (A), t is the electrolysis time (h), and product unit is the amount of the target product formed. EC quantifies the electrical energy required to produce one mole of product, incorporating the effects of overpotential, Faradaic efficiency, and product yield (i.e., conversion × selectivity). Lower EC values indicate greater energy efficiency.

Furthermore, the energy storage efficiency (ESE) was calculated to assess the extent to which the input electrical energy is stored in the chemical bonds of liquid fuel products:

$$\boldsymbol{ESE}\ (\%) = \sum\left(\frac{mol_{product} \times \Delta_{Combustion}H^0_{LA\rightarrow product}}{E \times I \times t/1000000}\right)\ \times 100$$

Here, $\Delta_{Combustion}H^0_{LA\rightarrow product}$ represents the standard combustion enthalpy gain for the hydrogenation of LA into the corresponding product: 0.23 MJ mol^{-1} for GVL (2.65 – 2.42 MJ mol^{-1}), 0.19 MJ mol^{-1} for HVA (2.61 – 2.42 MJ mol^{-1}), and 0.42 MJ mol^{-1} for VA (2.84 – 2.42 MJ mol^{-1}). A higher ESE indicates more efficient conversion of electrical energy into chemical energy, highlighting the potential of this system for renewable energy storage through the generation of stable, energy-dense liquid fuels.

Table S4: Electrocatalytic performance of CuNi-based catalysts (CuNi-25 and CuNi-50) for the electrochemical hydrogenation of levulinic acid (LA) at different temperatures (5–50 °C) and applied potentials (–1.6, –1.8, and –2.0 V vs. Ag|AgCl). For each condition, LA conversion (%), product distribution (GVL, HVA, VA, and other products (OP), all in mol%), Faradaic efficiency (FE, %), energy consumption (EC, in kWh mol^{-1}), and energy storage efficiency (ESE, %) are reported. CuNi-25 and CuNi-50 refer to catalysts electrodeposited at 25 and 50 mC cm^{-2}, respectively. GVL: γ-valerolactone; HVA: 4-hydroxypentanoic acid; VA: valeric acid.

T / ºC	CuNi-25																							
	–1.6 V								–1.8 V								–2.0 V							
	**Conversion / %**	**GVL / %**	**HVA / %**	**VA / %**	**OP / %**	**FE / %**	**EC / kWh mol^{-1}**	**ESE / %**	**Conversion / %**	**GVL / %**	**HVA / %**	**VA / %**	**OP / %**	**FE / %**	**EC / kWh mol^{-1}**	**ESE / %**	**Conversion / %**	**GVL / %**	**HVA / %**	**VA / %**	**OP / %**	**FE / %**	**EC / kWh mol^{-1}**	**ESE / %**
5	88.3	0.6	83.6	15.1	0.7	64.9	0.133	46.9	85.3	0.9	93.7	5.4	0.02	57.8	0.137	41.1	82.5	1.21	95.95	2.82	0.02	54.5	0.142	38.6
15	89.6	22.0	54.4	23.5	0.1	71.1	0.131	53.8	86.9	25.7	63.5	10.8	0.05	61.9	0.135	46.4	85.9	27.88	65.42	6.66	0.04	58.9	0.136	44.2
25	92.0	56.1	11.1	32.7	0.1	78.5	0.127	62.8	90.1	59.9	14.1	25.9	0.07	72.9	0.130	58.5	89.6	58.53	15.05	26.3	0.12	72.7	0.131	58.3
35	93.9	67.1	1.2	31.5	0.1	79.4	0.125	64.5	92.3	82.3	1.7	15.9	0.14	68.7	0.127	56.8	91.8	86.83	2.28	10.79	0.09	65.3	0.128	54.4
50	94.5	78.5	0.0	21.3	0.1	73.6	0.124	60.6	92.8	84.2	0.0	15.7	0.08	69	0.126	57.3	92.3	93.24	0.0	6.74	0.03	63.3	0.127	53.2

T / ºC	CuNi-50																							
	–1.6 V								–1.8 V								–2.0 V							
	**Conversion / %**	**GVL / %**	**HVA / %**	**VA / %**	**OP / %**	**FE / %**	**EC / kWh mol^{-1}**	**ESE / %**	**Conversion / %**	**GVL / %**	**HVA / %**	**VA / %**	**OP / %**	**FE / %**	**EC / kWh mol^{-1}**	**ESE / %**	**Conversion / %**	**GVL / %**	**HVA / %**	**VA / %**	**OP / %**	**FE / %**	**EC / kWh mol^{-1}**	**ESE / %**
5	92.1	0.8	86.6	12.6	0.01	66.7	0.127	48.0	88.0	1.1	93.8	5.11	0.02	59.5	0.133	42.3	83.1	2.06	96.8	1.2	0.01	54.1	0.141	38.2
15	93.3	20.5	58.1	21.3	0.07	72.7	0.125	54.7	90.3	23.0	67.6	9.32	0.04	63.5	0.130	47.3	86.8	21.4	72.3	6.2	0.04	59.3	0.135	43.9
25	93.5	58.5	11.0	30.4	0.09	78.3	0.125	62.9	91.6	62.0	14	23.9	0.10	73	0.128	58.7	89.9	69.1	18.1	12.7	0.11	65.1	0.130	52.7
35	95.9	53.5	1.7	44.7	0.15	89.2	0.122	71.5	94.1	64.6	2.22	33.1	0.16	80.4	0.124	65.2	92.6	72.7	3.2	23.9	0.17	73.7	0.127	60.2
50	96.5	68.6	0.0	31.2	0.16	81.4	0.121	66.3	94.5	76.0	0.0	23.9	0.11	75.2	0.124	61.8	93.0	84.9	0.0	15.1	0.01	68.9	0.126	57.2

Table S5: Electrocatalytic performance of CuNiRu-based catalysts (CuNi-25 and CuNi-50) for the electrochemical hydrogenation of levulinic acid (LA) at different temperatures (5–50 °C) and applied potentials (–1.6, –1.8, and –2.0 V vs. Ag|AgCl). For each condition, LA conversion (%), product distribution (GVL, HVA, VA, and other products (OP), all in mol%), Faradaic efficiency (FE, %), energy consumption (EC, in kWh mol^{-1}), and energy storage efficiency (ESE, %) are reported. CuNi-25 and CuNi-50 refer to catalysts electrodeposited at 25 and 50 mC cm^{-2}, respectively. GVL: γ-valerolactone; HVA: 4-hydroxypentanoic acid; VA: valeric acid.

	CuNiRu-25																							
T / ºC	–1.6 V								–1.8 V								–2.0 V							
	**Conversion / %**	**GVL / %**	**HVA / %**	**VA / %**	**OP / %**	**FE / %**	**EC / kWh mol^{-1}**	**ESE / %**	**Conversion / %**	**GVL / %**	**HVA / %**	**VA / %**	**OP / %**	**FE / %**	**EC / kWh mol^{-1}**	**ESE / %**	**Conversion / %**	**GVL / %**	**HVA / %**	**VA / %**	**OP / %**	**FE / %**	**EC / kWh mol^{-1}**	**ESE / %**
5	89.2	0.7	93.0	6.3	0.0	61	0.131	43.4	86.3	0.9	95.1	4.0	0.0	57.7	0.135	40.9	84.1	1.3	97.5	1.2	0.0	54.7	0.139	38.6
15	91.5	26.1	70.7	3.2	0.0	60.7	0.128	45.2	89.2	28.5	64.3	7.2	0.0	61.5	0.131	46.2	87.7	30.3	66.4	3.3	0.0	58.3	0.133	43.7
25	93.7	61.9	13.8	24.2	0.1	74.8	0.125	60.2	92.1	67.9	13.7	18.3	0.1	70	0.127	56.7	91.1	69.8	14.7	15.4	0.1	67.6	0.128	54.8
35	94.6	77.8	1.3	20.8	0.2	73.4	0.124	60.4	92.7	86.4	2.5	11.0	0.1	66.1	0.126	55.0	92.5	92.9	1.7	5.4	0.0	62.7	0.126	52.7
50	95.1	87.6	0.0	12.3	0.1	68.6	0.123	57.2	93.2	93.4	0.0	6.6	0.1	63.9	0.125	53.7	93.1	97.5	0.0	2.4	0.0	61.3	0.126	51.8

	CuNiRu-50																							
T / ºC	–1.6 V								–1.8 V								–2.0 V							
	**Conversion / %**	**GVL / %**	**HVA / %**	**VA / %**	**OP / %**	**FE / %**	**EC / kWh mol^{-1}**	**ESE / %**	**Conversion / %**	**GVL / %**	**HVA / %**	**VA / %**	**OP / %**	**FE / %**	**EC / kWh mol^{-1}**	**ESE / %**	**Conversion / %**	**GVL / %**	**HVA / %**	**VA / %**	**OP / %**	**FE / %**	**EC / kWh mol^{-1}**	**ESE / %**
5	92.9	0.9	95.1	4	0	62.1	0.126	44.0	89.6	1.2	96.4	2.4	0	59	0.130	41.7	85.4	1.5	97.7	0.8	0.0	55.4	0.137	39.0
15	94.4	26.2	72.1	1.7	0	61.8	0.124	45.8	91.7	28.5	66.9	4.6	0	61.7	0.127	46.2	88.4	32.6	64.2	3.3	0.0	58.8	0.132	44.3
25	95.6	61.6	12.8	25.5	0.1	77.1	0.122	62.0	93.7	65.9	13.1	20.9	0.1	72.8	0.125	58.8	91.6	67	14.9	18	0.1	69.5	0.128	56.2
35	96.1	70.7	1.2	27.9	0.2	78.9	0.122	64.4	95.1	82.9	2.1	14.9	0.1	70.2	0.123	58.1	93.2	94.6	1.7	3.7	0.0	62.2	0.125	52.3
50	96.6	75.2	0.0	24.7	0.1	77.4	0.121	63.5	95.8	88.2	0.0	11.7	0.1	68.8	0.122	57.4	93.8	98.5	0.0	1.4	0.0	61.1	0.125	51.8

Table S6: Comparison of CuNiRu/GNW performance with representative Pb-, Cd-, In-, Ni-, Pt- and carbon-based electrocatalysts for LA reduction, including conversion, main product, selectivity and FE.

Catalyst	*Conditions*	*Conversion / %*	*Main product*	*Selectivity / %*	*FE / %*	*Reference*
Pb (bulk)	0.1 M $KHCO_3$ + 0.1 M $KClO_4$, −1.9 V vs RHE, 50 °C	97.0	GVL	93.0	65	[1]
Fe sheet (1 cm^2)	0.5 M H_2SO_4	19.9	VA	9.6	0.5	[2]
Cu sheet (1 cm^2)	0.5 M H_2SO_4	7.3	GVL	9.6	1.3	[3]
C sheet (1 cm^2)	0.5 M H_2SO_4	39.0	GVL	69.0	18.0	[3]
Cd wire (1 mm)	1 M H_2SO_4 + 0.5 M LA	98.0	VA	91.1	44.8	[4]
Pb wire (1 mm)	1 M H_2SO_4 + 0.5 M LA	98.6	VA	94.0	46.1	[4]
In wire (1 mm Ø)	1 M H_2SO_4 + 0.5 M LA	88.4	VA	99.1	43.3	[4]
Ni sheet (1 cm^2)	0.5 M H_2SO_4	8.0	VA	17.3	1.1	[4]
Fe sheet (1 cm^2)	1 M KOH	24.5	HVA	18.5	74.5	[5]
Pb sheet (1 cm^2)	1 M KOH	71.6	HVA	81.6	18.4	[5]
Pb plate	Flow cell; −1.3 V vs RHE, 0.2 M LA (H_2SO_4)	18.6	GVL	95.0	86.5	[5]
Pb plate	Flow cell; −1.9 V vs RHE, 0.1 M $KHCO_3$ + $KClO_4$	92.0	GVL	93.0	47.1	[5]
Cu CFs	0.1 M H_2SO_4 (2 h)	41.0	VA	13.2	31.1	[5]
Ni CFs	0.1 M H_2SO_4 (2 h)	13.5	GVL	50.8	6.7	[5]
Pt CFs	0.1 M H_2SO_4 (2 h)	12.1	GVL	35.9	8.9	[5]
Pb wires	1 M H_2SO_4, 50 °C	98.0	VA	94.0	44.6	[5]
PbS	Et_4NBF_4 ionic liquid, −2.15 V vs Ag|AgCl, 4 h	—	GVL	100	78.6	[5]
β-PbO/Pb	0.5 M H_2SO_4, −1.4 V vs RHE, 4 h	≈100	VA	95.0	57.6	[5]

References

[1] F. W. S. Lucas, Y. Fishler, A. Holewinski, *Green Chem.* **2021**, *23*, 9154.

[2] P. Nilges, T. R. Dos Santos, F. Harnisch, U. Schröder, *Energy Environ. Sci.* **2012**, *5*, 5231.

[3] T. R. Dos Santos, P. Nilges, W. Sauter, F. Harnisch, U. Schröder, *RSC Adv.* **2015**, *5*, 26634.

[4] R. J. M. Bisselink, M. Crockatt, M. Zijlstra, I. J. Bakker, E. Goetheer, T. M. Slaghek, D. S. van Es, *ChemElectroChem* **2019**, *6*, 3285.

[5] Y. Zhang, Y. Shen, *Appl. Catal. B Environ.* **2024**, *343*, 123576.
